# Performance evaluation of pipelines for mapping, variant calling and interval padding, for the analysis of NGS germline panels

**DOI:** 10.1186/s12859-021-04144-1

**Published:** 2021-04-28

**Authors:** Maria Zanti, Kyriaki Michailidou, Maria A. Loizidou, Christina Machattou, Panagiota Pirpa, Kyproula Christodoulou, George M. Spyrou, Kyriacos Kyriacou, Andreas Hadjisavvas

**Affiliations:** 1 Department of Electron Microscopy/Molecular Pathology, The Cyprus Institute of Neurology and Genetics, 2371 Nicosia, Cyprus; 2 Cyprus School of Molecular Medicine, 2371 Nicosia, Cyprus; 3 Bioinformatics Department, The Cyprus Institute of Neurology and Genetics, 2371 Nicosia, Cyprus; 4 Biostatistics Unit, The Cyprus Institute of Neurology and Genetics, 2371 Nicosia, Cyprus; 5 Neurogenetics Department, The Cyprus Institute of Neurology and Genetics, 2371 Nicosia, Cyprus

**Keywords:** Next-generation sequencing (NGS), Germline NGS data analysis, Variant calling, Alignment, Interval padding, Pipeline comparison

## Abstract

**Background:**

Next-generation sequencing (NGS) represents a significant advancement in clinical genetics. However, its use creates several technical, data interpretation and management challenges. It is essential to follow a consistent data analysis pipeline to achieve the highest possible accuracy and avoid false variant calls. Herein, we aimed to compare the performance of twenty-eight combinations of NGS data analysis pipeline compartments, including short-read mapping (BWA-MEM, Bowtie2, Stampy), variant calling (GATK-HaplotypeCaller, GATK-UnifiedGenotyper, SAMtools) and interval padding (null, 50 bp, 100 bp) methods, along with a commercially available pipeline (BWA Enrichment, Illumina®). Fourteen germline DNA samples from breast cancer patients were sequenced using a targeted NGS panel approach and subjected to data analysis.

**Results:**

We highlight that interval padding is required for the accurate detection of intronic variants including spliceogenic pathogenic variants (PVs). In addition, using nearly default parameters, the BWA Enrichment algorithm, failed to detect these spliceogenic PVs and a missense PV in the *TP53* gene. We also recommend the BWA-MEM algorithm for sequence alignment, whereas variant calling should be performed using a combination of variant calling algorithms; GATK-HaplotypeCaller and SAMtools for the accurate detection of insertions/deletions and GATK-UnifiedGenotyper for the efficient detection of single nucleotide variant calls.

**Conclusions:**

These findings have important implications towards the identification of clinically actionable variants through panel testing in a clinical laboratory setting, when dedicated bioinformatics personnel might not always be available. The results also reveal the necessity of improving the existing tools and/or at the same time developing new pipelines to generate more reliable and more consistent data.

**Supplementary Information:**

The online version contains supplementary material available at 10.1186/s12859-021-04144-1.

## Background

Massively parallel sequencing, also known as next-generation sequencing (NGS), represents a significant advancement in clinical genetics and has revolutionized the field of molecular genetics, as it enables the investigation of several genes and samples simultaneously [1]. To this end, massively parallel sequencing, has set the ground for the discovery of novel disease causative variants [2]. However, these newly integrated technologies are accompanied with several technical, data management and interpretation challenges [3]. Although a diversity of sequence mapping and variant calling methods have been developed, they present variable concordance between their calls [3–12].

Among many short-read mapping algorithms, Burrows–Wheeler Aligner (BWA)-Maximal Exact Match (MEM) [13], Stampy [14] and Bowtie2 [15] are very popular. Bowtie2 and BWA-MEM use the Burrows-Wheeler transform (BWT) algorithm, during which the reference genome is “collapsed” and indexed and reads are aligned against substrings of the reference genome [16, 17]. Subsequently, both produce very similar results [13, 18]. In contrast, Stampy which uses a hash-based approach by hashing the reference genome in 15-mers, identifies candidate alignment locations for each read in the hash table, which are then filtered to discover the sequence with the highest read similarity [14].

Through the years, divergent variant calling algorithms have been developed which function by distinguishing true variants from alignment errors [10]. The Genome Analysis ToolKit (GATK)-HaplotypeCaller (GATK-HC) [19], GATK-UnifiedGenotyper (GATK-UG) [20] and SAMtools [21] variant calling algorithms are widely used. GATK-UnifiedGenotyper and SAMtools, follow a Bayesian variant calling approach to model sequencing errors and detect candidate variants by independently mapping reads to the reference genome and evaluating genotype likelihoods to model sequencing errors and identify the most likely genotype call [10–12, 22]. This approach can be very efficient for the detection of single nucleotide variants (SNVs), but may pose challenges when aligning reads to regions surrounding candidate insertions or deletions (indels) [10]. On the other hand, GATK-HaplotypeCaller follows an assembly-based approach, during which it first carries out a local de-novo assembly of reads within a fixed-length window, then builds up candidate haplotypes and determines their likelihoods comparing to the reference genome [10]. Candidate haplotypes with the highest likelihood are those called as true sequences and variants within the haplotype are then called as true variants. This assembly-based approach can be more efficient for the detection of small or even large indels, since it can address incorrect alignments in regions beside candidate indels and thus improve the total accuracy and recall compared to Bayesian variant-calling approaches [10].

In addition, variant calling requires an interval list file, which corresponds to the genomic regions targeted during library preparation and is typically provided by the kit manufacturer. For exome or targeted sequencing data, the GATK (https://gatk.broadinstitute.org/) suite recently suggested additional interval padding (usually 100 bp). Although interval padding is clearly stated as an optional parameter in the documentation of various variant calling algorithms and indeed interval padding is being used [23], variant calling algorithms running with nearly default parameters may miss potentially actionable spliceogenic pathogenic variants (PVs), while some algorithms do not include options on that parameter.

Following good laboratory standards for clinical NGS [22], we included positive controls in each run and noticed that data analysis following the GATK best practice guidelines, led to low detection rates of the known PVs. Due to substantial performance variation among different pipelines, the EuroGentest project and the European Society of Human Genetics, proposed guidelines for the evaluation and validation of NGS applications for the diagnosis of genetic disorders [24]. The ultimate goal is to define the most appropriate pipeline for each technology, achieve the highest possible accuracy and minimize false variant calls.

Herein, we aimed to compare the variant calling performance of twenty-eight combinations of pipeline compartments, including three short-read alignment algorithms—BWA-MEM, Bowtie2 and Stampy—, three variant calling algorithms—GATK-HC, GATK-UG and SAMtools—and three different interval padding lengths (null, 50 bp and 100 bp), as well as a commercially available pipeline (BWA Enrichment, Illumina®).

## Results

### Sequencing and mapping evaluation

Sequencing was performed on the NextSeq 500 Sequencing Platform (Illumina) using high-output v2.5 kits with 2 × 75 or 2 × 150 cycles. Both runs obtained high quality scores (Q-score). Q30 rate was achieved for 87.8% (> 80%) and 78.6% (> 75%) of reads, while cluster density was at optimal levels (on average 215 k/mm^2^ and 210 k/mm^2^, respectively). The output yield was relatively high (77.9Gbp and 128.8Gbp). Sequencing, generated an average of 20.6 and 14.8 million reads, whereas clusters generated were on average 10.3 and 7.4 for each 2 × 75 and 2 × 150 run respectively (Additional file 3).

**Fig. 1:**
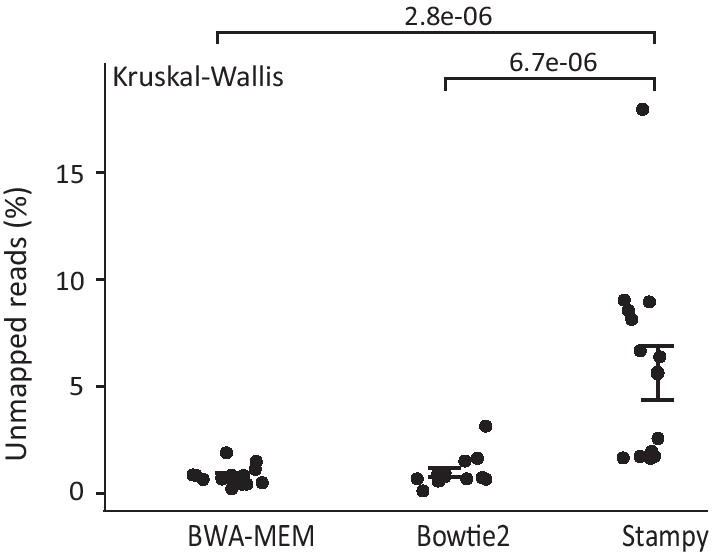
Evaluation of mapping efficiency per alignment algorithm. Dot plot showing the distribution of unmapped reads (percentage). Standard deviation values are shown in error bars. Detailed numbers are shown in Additional file 4. Statistical analysis was performed using the non-parametric Kruskal–Wallis test

All reads were mapped to the hg19 reference human genome assembly (GRCh37) (https://genome.ucsc.edu/, last accessed 19/07/2019) and more than 99% of reads were properly aligned to the reference genome. However, as shown in Fig. 1, the mapping efficiency of Stampy was lower compared to the other two aligners. In detail, alignment with Stampy demonstrated a higher number of unmapped reads compared to BWA-MEM and Bowtie2; a trend that applied to all samples included in the study (Fig. 1, Additional file 3). On average, Stampy failed to align 5.622% of reads, compared to 0.810% and 0.967% of reads by BWA-MEM and Bowtie2 respectively (*p* value = 2.80 × 10^–06^, *p* value = 6.70 × 10^–06^, Kruskal–Wallis) (Fig. 1, Additional file 4). Although, both BWA-MEM and Bowtie2 algorithms accomplished high mapping efficiencies, the BWA-MEM tool possessed the highest mapping power (99.189% of generated reads were mapped) (Additional file 4). However, the difference was not statistically significant (*p* value = 1, Kruskal–Wallis).

The GATK DepthOfCoverage tool was used to examine the depth of coverage (DP) for the 18 genes under investigation. Detailed maximum, mean and minimum DP values per gene, are shown in Additional file 5. As expected, sequencing with 2 × 75 cycles, resulted in about half DP compared to sequencing with 2 × 150 cycles (Additional file 5). Alignment with BWA-MEM resulted on an average of 385 reads per base (X) DP (range: 2–1188) and 735X DP (range: 21–2452) corresponding to sequencing with 2 × 75 and 2 × 150 cycles, respectively. Mapping with Bowtie2, demonstrated 385 (range: 4–1201) and 739 (range 25–2459) DP, corresponding to 2 × 75 and 2 × 150 cycles sequencing, respectively. In addition, mapping with Stampy, demonstrated a lower DP compared to others; 382 (range 4–1187) and 699 (range 16–2425), corresponding to sequencing with 2 × 75 and 2 × 150 cycles, respectively. Hence, sequencing with 300 cycles and mapping with BWA-MEM or Bowtie2 resulted to a higher on average DP.

Following variant filtering, we applied a cut-off value of DP ≥ 30X. For all samples and alignment methods, sequence reads sufficiently covered more than 99% of the targeted regions. Bowtie2 alignment on 300 cycles sequencing data, demonstrated the highest coverage performance (~ 100%) which is close to the coverage performance of BWA-MEM and Stampy methods (99.982% and 99.963%, respectively). Only two regions demonstrated low (< 30X) DP; the splice donor site of exon 1 of the *MSH6* gene and 23 nucleotides residing at the end of exon 5 of the *MSH2* gene. Despite the lower mapping power, alignment with Stampy, demonstrated a slightly higher coverage performance (99.547%) on alignment of 2 × 75 cycles sequencing data, compared to BWA-MEM and Bowtie (99.522% and 99.527%, respectively). Twenty-three intervals demonstrated low (< 30X) DP, of which the less covered (< 30X for more than 10% of the exon region) were the *STK11*_exon7, *STK11*_exon4, *NF1*_exon 25, *ATM*_exon43, *MSH2*_exon5, *NF1*_exon14 and *NF1*_exon30 (Additional file 5).

### Pipeline comparison and ranking

**Fig. 2:**
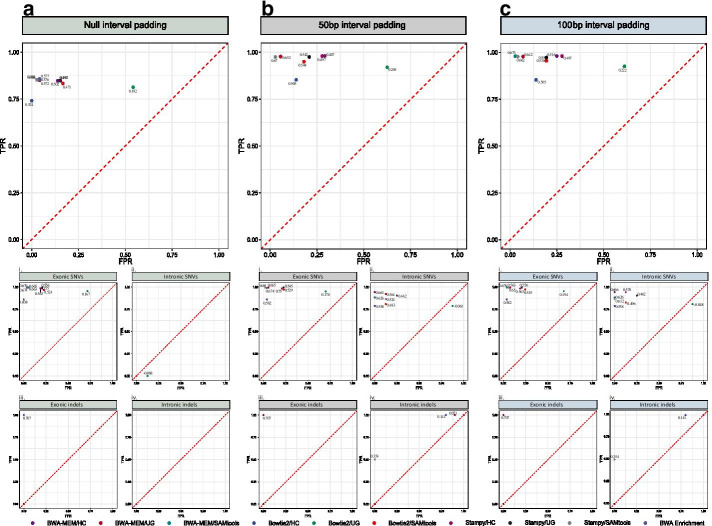
Receiver-operating characteristic space plots comparing variant calls. ROC space comparing variant calls upon **a** null interval padding, **b** 50 bp interval padding and **c** 100 bp interval padding for the entity of variants (top panel), divided to exonic SNVs (Ai/Bi/Ci), intronic SNVs (Aii/Bii/Cii), exonic indels (Aiii/Biii/Ciii) and intronic indels (Aiv/Biv/Civ). Sanger Sequencing was used as the gold standard to evaluate the accuracy of the calls. True and false positive rates were plotted in the Receiver Operating Characteristic (ROC) space. Each point corresponds to an instance of a confusion matrix. Labels correspond to perpendicular distance (*d*) values. *FPR* false positive rate, *GATK* Genome Analysis ToolKit, *HC* HaplotypeCaller, *TPR* true positive rate, *UG* UnifiedGenotyper. TPR and FPR stand for true positive and false positive rates. The “Random Guess” line is shown in red

**Fig. 3:**
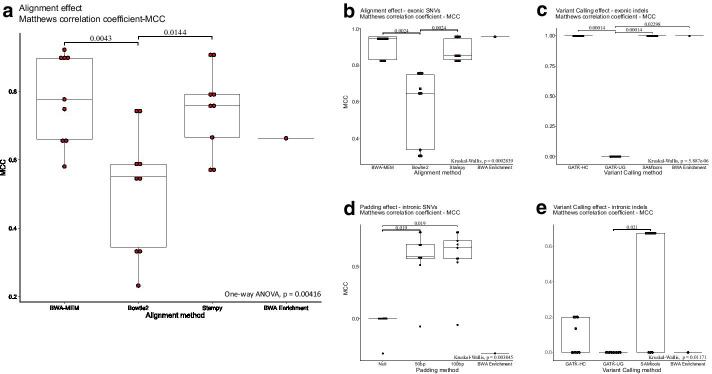
Boxplots illustrating the Matthews correlation coefficient values by alignment, variant calling or padding method. **a** Entity of variants. **b** exonic SNVs. **c** exonic indels. **d** intronic SNVs. **e** intronic indels. Each dot represents one observation and horizontal bold lines denote median MCC values. Boxes extend from the 25th to the 75th percentile of each group’s distribution of values. Vertical extending lines (whiskers) denote the upper and lower adjacent values. Statistical analyses were performed using the non-parametric Kruskal–Wallis or one-way ANOVA tests. All box plots of MCC values, including not statistically significant correlations, are shown in Additional file 7. *GATK* Genome Analysis ToolKit, *HC* HaplotypeCaller, *UG* UnifiedGenotyper

We evaluated the performance of each of the pipelines using data from the 14 samples. Ranking was carried out using the perpendicular distance (*d*) of each point from the “Random Guess”, the so-called no discrimination, diagonal line (Fig. 2). At first, pipeline performance was compared for all variant types. BWA-MEM/SAMtools with 100 bp padding, demonstrated the highest overall performance, followed by BWA-MEM/SAMtools and Stampy/SAMtools pipelines with 50 bp padding (Fig. 2). The corresponding (*d*) values were 0.673, 0.670 and 0.670, respectively (Fig. 2). Stampy/SAMtools with 100 bp padding, along with BWA-MEM/GATK-UG with 50 bp padding, ranked next, with perpendicular (*d*) values 0.662 and 0.652. We observed similar results when comparing Matthews correlation coefficient (MCC) and F1 scores (Table 1). Detailed numbers of true positive and false positive SNVs and indels, are provided in Table 2. The Illumina BWA Enrichment application demonstrated a 0.577 perpendicular (*d*) value, which is slightly higher, compared to the overall performance of null padding pipelines (*d* = 0.489). Hierarchical clustering based on perpendicular (*d*), MCC, F1 score, precision (p) and recall (r) metrics supports our observations (Table 1, Additional file 6). Statistical association tests demonstrated that the read mapping method is statistically significant in association with total variant calling performance (*p* value = 0.00416, one-way ANOVA). In detail, Bowtie2 (MCC = 0.517) exhibited reduced performance compared to BWA-MEM (MCC = 0.782, *p* value = 0.0043) and Stampy (MCC = 0.747, *p* value = 0.0144) (Fig. 3a).

**Table 1:** Matthews correlation coefficient, perpendicular distance, true positive rate and false positive rate values

	Exonic SNVs				Exonic indels				Intronic SNVs			
	MCC	d	TPR	FPR	MCC	d	TPR	FPR	MCC	d	TPR	FPR
*Null* *interval* *padding*												
BWA-MEM/GATK-HC	0.832	0.563	0.985	0.189	**1.000**	0.707	1.000	0.000	0.000	0.000	0.000	0.000
BWA-MEM/GATK-UG	**0.956**	0.665	0.997	0.057	0.000	0.000	0.000	0.000	0.000	0.000	0.000	0.000
BWA-MEM/SAMtools	0.945	0.663	0.994	0.057	**1.000**	0.707	1.000	0.000	0.000	0.000	0.000	0.000
Bowtie2/GATK-HC	0.672	0.608	0.860	0.000	**1.000**	0.707	1.000	0.000	0.000	0.000	0.000	0.000
Bowtie2/GATK-UG	0.300	0.167	0.953	0.717	0.000	0.000	0.000	0.000	− 0.332	− 0.088	0.000	0.125
Bowtie2/SAMtools	0.748	0.524	0.968	0.226	**1.000**	0.707	1.000	0.000	0.000	0.000	0.000	0.000
Stampy/GATK-HC	0.819	0.550	0.985	0.208	**1.000**	0.707	1.000	0.000	0.000	0.000	0.000	0.000
Stampy/GATK-UG	0.852	0.556	0.994	0.208	0.000	0.000	0.000	0.000	0.000	0.000	0.000	0.000
Stampy/SAMtools	0.946	0.674	0.991	0.038	**1.000**	0.707	1.000	0.000	0.000	0.000	0.000	0.000
*50* *bp* *interval* *padding*												
BWA-MEM/GATK-HC	0.819	0.550	0.985	0.208	**1.000**	0.707	1.000	0.000	**0.828**	0.666	0.942	0.000
BWA-MEM/GATK-UG	**0.956**	0.665	0.997	0.057	0.00	0.000	0.000	0.000	0.701	0.564	0.923	0.125
BWA-MEM/SAMtools	0.946	0.674	0.991	0.038	**1.000**	0.707	1.000	0.000	0.711	0.626	0.885	0.000
Bowtie2/GATK-HC	0.647	0.582	0.860	0.038	**1.000**	0.707	1.000	0.000	0.576	0.558	0.789	0.000
Bowtie2/GATK-UG	0.311	0.178	0.950	0.698	0.000	0.000	0.000	0.000	− 0.074	− 0.061	0.789	0.875
Bowtie2/SAMtools	0.767	0.529	0.974	0.226	**1.000**	0.707	1.000	0.000	0.515	0.483	0.808	0.125
Stampy/GATK-HC	0.819	0.550	0.985	0.208	**1.000**	0.707	1.000	0.000	**0.828**	0.666	0.942	0.000
Stampy/GATK-UG	0.852	0.545	0.997	0.226	0.000	0.000	0.000	0.000	0.574	0.462	0.904	0.250
Stampy/SAMtools	**0.968**	0.690	0.994	0.019	**1.000**	0.707	1.000	0.000	0.595	0.524	0.865	0.125
*100* *bp* *interval* *padding*												
BWA-MEM/GATK-HC	0.831	0.563	0.985	0.189	**1.000**	0.707	1.000	0.000	**0.828**	0.666	0.942	0.000
BWA-MEM/GATK-UG	0.934	0.650	0.994	0.075	0.000	0.000	0.000	0.000	0.746	0.578	0.942	0.125
BWA-MEM/SAMtools	**0.956**	0.676	0.994	0.038	**1.000**	0.707	1.000	0.000	0.711	0.626	0.885	0.000
Bowtie2/GATK-HC	0.647	0.582	0.860	0.038	**1.000**	0.707	1.000	0.000	0.576	0.558	0.789	0.000
Bowtie2/GATK-UG	0.338	0.194	0.953	0.679	0.000	0.000	0.000	0.000	− 0.059	− 0.048	0.808	0.875
Bowtie2/SAMtools	0.753	0.515	0.974	0.245	**1.000**	0.707	1.000	0.000	0.540	0.496	0.827	0.125
Stampy/GATK-HC	0.831	0.563	0.985	0.189	**1.000**	0.707	1.000	0.000	**0.828**	0.666	0.942	0.000
Stampy/GATK-UG	0.852	0.556	0.994	0.208	0.000	0.000	0.000	0.000	0.574	0.462	0.904	0.250
Stampy/SAMtools	0.945	0.663	0.994	0.057	**1.000**	0.707	1.000	0.000	0.679	0.612	0.865	0.000
BWA Enrichment	**0.956**	0.676	0.994	0.038	**1.000**	0.707	1.000	0.000	− 0.331	− 0.088	0.000	0.1250

	Intronic indels				All types of variants			
	MCC	d	TPR	FPR	MCC	d	TPR	FPR
*Null* *interval* *padding*								
BWA-MEM/GATK-HC	0.000	0.000	0.000	0.000	0.581	0.502	0.848	0.139
BWA-MEM/GATK-UG	0.000	0.000	0.000	0.000	0.653	0.572	0.851	0.042
BWA-MEM/SAMtools	0.000	0.000	0.000	0.000	0.660	0.576	0.856	0.042
Bowtie2/GATK-HC	0.000	0.000	0.000	0.000	0.551	0.524	0.741	0.000
Bowtie2/GATK-UG	0.000	0.000	0.000	0.000	0.233	0.192	0.813	0.542
Bowtie2/SAMtools	0.000	0.000	0.000	0.000	0.540	0.471	0.833	0.167
Stampy/GATK-HC	0.000	0.000	0.000	0.000	0.571	0.492	0.848	0.153
Stampy/GATK-UG	0.000	0.000	0.000	0.000	0.571	0.492	0.848	0.153
Stampy/SAMtools	0.000	0.000	0.000	0.000	0.666	0.584	0.853	0.028
*50* *bp* *interval* *padding*								
BWA-MEM/GATK-HC	0.000	0.000	1.000	1.000	0.749	0.487	0.980	0.292
BWA-MEM/GATK-UG	0.000	0.000	0.000	0.000	**0.898**	**0.652**	0.978	0.056
BWA-MEM/SAMtools	**0.674**	0.354	0.500	0.000	**0.908**	**0.670**	0.975	0.028
Bowtie2/GATK-HC	0.200	0.414	1.000	0.800	0.588	0.505	0.853	0.139
Bowtie2/GATK-UG	0.000	0.000	0.000	0.000	0.321	0.209	0.920	0.625
Bowtie2/SAMtools	**0.674**	0.354	0.500	0.000	0.741	0.544	0.950	0.181
Stampy/GATK-HC	0.135	0.071	1.000	0.900	0.758	0.497	0.980	0.278
Stampy/GATK-UG	0.000	0.000	0.000	0.000	0.790	0.542	0.975	0.208
Stampy/SAMtools	**0.674**	0.354	0.500	0.000	**0.908**	**0.670**	0.975	0.028
*100* *bp* *interval* *padding*								
BWA-MEM/GATK-HC	0.200	0.414	1.000	0.800	0.777	0.516	0.980	0.250
BWA-MEM/GATK-UG	0.00	0.000	0.000	0.000	0.888	0.642	0.978	0.069
BWA-MEM/SAMtools	**0.674**	0.354	0.500	0.000	**0.922**	**0.673**	0.980	0.028
Bowtie2/GATK-HC	0.200	0.414	1.000	0.800	0.588	0.505	0.853	0.139
Bowtie2/GATK-UG	0.000	0.000	0.000	0.000	0.344	0.222	0.925	0.611
Bowtie2/SAMtools	**0.674**	0.354	0.500	0.000	0.744	0.538	0.955	0.194
Stampy/GATK-HC	0.000	0.000	1.000	1.000	0.758	0.497	0.980	0.278
Stampy/GATK-UG	0.000	0.000	0.000	0.000	0.792	0.550	0.973	0.194
Stampy/SAMtools	**0.674**	0.354	0.500	0.000	**0.906**	**0.662**	0.978	0.042
BWA Enrichment	0.000	0.000	0.000	0.000	0.663	0.577	0.858	0.042

For pipeline performance evaluation, variants were categorized in four groups; exonic single nucleotide variants (SNVs) and exonic indels (insertions or deletions), intronic SNVs (± 1- ± 10) and intronic indels (± 1– ± 10). Sanger sequencing was performed to validate the calls. *d* perpendicular distance, *GATK* Genome Analysis ToolKit, *FPR* false positive rate, *HC* HaplotypeCaller; indels, insertions & deletions; *MCC* Matthews correlation coefficient, *SNVs* single nucleotide variants, *TPR* true positive rate, *UG* UnifiedGenotyper; in bold, selected top tier performing pipelines as per MCC ranking

**Table 2:** Numbers of true positive and false positive single nucleotide variants and insertions/deletions, detected by each pipeline combination

	True positive SNVs	False positive SNVs	True positive indels	False positive indels
*Null* *interval* *padding*				
BWA-MEM/GATK-HC	338	10	3	0
BWA-MEM/GATK-UG	342	3	0	0
BWA-MEM/SAMtools	341	3	3	0
Bowtie2/GATK-HC	295	0	3	0
Bowtie2/GATK-UG	327	39	0	0
Bowtie2/SAMtools	332	12	3	0
Stampy/GATK-HC	338	11	3	0
Stampy/GATK-UG	341	11	0	0
Stampy/SAMtools	340	2	3	0
*50* *bp* *interval* *padding*				
BWA-MEM/GATK-HC	387	12	5	9
BWA-MEM/GATK-UG	390	4	0	0
BWA-MEM/SAMtools	386	2	4	0
Bowtie2/GATK-HC	336	3	5	7
Bowtie2/GATK-UG	367	44	0	0
Bowtie2/SAMtools	376	13	4	0
Stampy/GATK-HC	387	12	5	8
Stampy/GATK-UG	389	14	0	0
Stampy/SAMtools	386	2	4	0
*100* *bp* *interval* *padding*				
BWA-MEM/GATK-HC	387	11	5	7
BWA-MEM/GATK-UG	390	5	0	0
BWA-MEM/SAMtools	387	2	4	0
Bowtie2/GATK-HC	336	3	5	7
Bowtie2/GATK-UG	369	43	0	0
Bowtie2/SAMtools	377	14	4	0
Stampy/GATK-HC	387	11	5	9
Stampy/GATK-UG	388	13	0	0
Stampy/SAMtools	386	3	4	0
BWA enrichment	341	3	3	0

For pipeline performance evaluation, variants were categorized in four groups; exonic single nucleotide variants (SNVs) and exonic indels (insertions or deletions), intronic SNVs (± 1 to ± 10) and intronic indels (± 1– ± 10). Sanger sequencing was performed to validate the calls. *GATK* Genome Analysis ToolKit, *HC* HaplotypeCaller; indels, insertions & deletions, *SNVs* single nucleotide variants, *UG* UnifiedGenotyper

The MCC values were used to rank the top tier performing pipeline combinations and perform analysis of variance for alignment, variant calling and padding methods. Stampy/SAMtools with 50 bp padding performed best on calling exonic single nucleotide variants (SNVs) with MCC = 0.968, followed by BWA-MEM/GATK-UG with zero padding, BWA-MEM/GATK-UG with 50 bp padding, BWA-MEM/SAMtools with 100 bp padding and BWA Enrichment application, all with MCC = 0.957 (Table 1). Statistical tests demonstrated that read mapping affects the exonic SNV calling performance (*p* value = 0.0002839, Kruskal–Wallis). In details, Bowtie2 (MCC = 0.576) exhibited reduced performance compared to BWA-MEM (MCC = 0.908, *p* value = 0.0024) and Stampy (MCC = 0.876, *p* value = 0.0024) (Fig. 3b). All GATK-UG based pipelines demonstrated deficient calling of exonic (*p* value = 5.9 × 10^–06^, Kruskal–Wallis) and intronic indels (*p* value = 0.01171, Kruskal–Wallis) (Fig. 3c, e, Table 1) irrespective of interval padding. It is noteworthy that all SAMtools based pipelines with 50 bp and 100 bp interval padding demonstrated the highest intronic indel calling performance (MCC = 0.6742). Towards intronic SNV calling, Stampy and BWA-MEM mapping, in combination with GATK-HC variant calling with 50 or 100 bp padding, demonstrated the highest performance (MCC = 0.828) (Table 1), followed by BWA-MEM/GATK-UG with 100 bp padding (MCC = 0.746). Statistical analyses demonstrated that the padding method affects the intronic SNV calling performance (*p* value = 0.003845, Kruskal–Wallis). In detail, 50 bp (MCC = 0.584) and 100 bp interval padding (MCC = 0.603) exhibited increased performance compared to null interval padding (MCC = −0.0369, *p* value = 0.019) (Fig. 3d). Detailed analyses of MCC variance for alignment, variant calling and padding methods are shown in Additional file 7.

### Detection of actionable variants

**Fig. 4:**
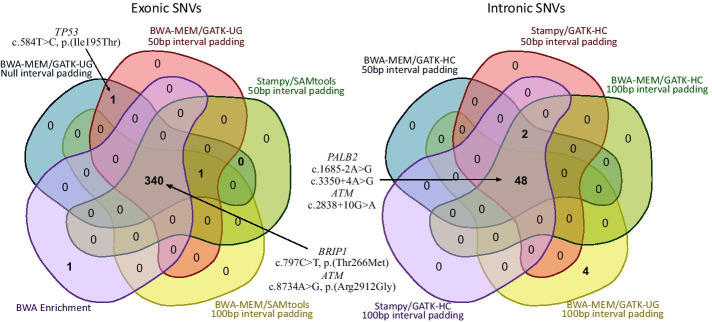
Venn diagrams depicting exonic and intronic true positive single nucleotide variants called by the top performing pipelines for exonic and intronic single nucleotide variants, respectively. Sanger sequencing was performed to validate the calls. Top tier performing pipelines were selected following the Matthews correlation coefficient ranking. Actionable variants fall in the groups that are depicted with arrows. *GATK* Genome Analysis ToolKit, *HC* HaplotypeCaller, *UG* UnifiedGenotyper

The top tier performing pipelines were selected based on the corresponding MCC value and compared for their concordance for SNV calls. As shown in Fig. 4, 99.13% (340/343) and 88.89% (48/54) of true positive exonic and intronic SNVs were called by all top-performing pipelines. Seven out of fourteen samples carried PVs in established breast cancer (BC) susceptibility genes. Among these, three patients carried splice-site PVs in the high penetrance susceptibility gene *PALB2* [c.1685-2A > G and c.3350 + 4A > G]. These, were only detected upon inclusion of interval padding (50 or 100 bp). Likewise, the BWA Enrichment application failed to detect these PVs. Hence, it appears that null padding and analysis with the BWA Enrichment application (Illumina), result in low detection rates of spliceogenic PVs. Three additional samples carried frameshift PVs in the *BRCA1* [c.1700dup, p.(Asn567fs)], *BRCA2* [c.3530_3533del, p.(Asp1177fs)] and *PALB2* [c.487_488del, p.(Val163fs)] genes, respectively. These frameshift PVs were detected by all pipeline combinations except those based on GATK-UG calling, irrespective of interval padding. In addition, one patient carried a missense *TP53* [c.584 T > C, p.(Ile195Thr)] PV. Although all pipeline combinations achieved the detection of the PV, this call was filtered out, subsequent to the variant allele frequency (VAF) threshold (≥ 30%). Only BWA-MEM/GATK-UG with null padding (VAF = 31.405%), BWA-MEM/GATK-UG with 50 bp padding (VAF = 30.579%) (Fig. 4) and Stampy/GATK-UG with 50 bp padding (VAF = 30.204%) pipelines, achieved to detect the missense PV with adequate VAF. Three more patients, carried missense variants of uncertain clinical significance (VUSs) (Fig. 4). Of these, two carried VUSs in the *ATM* [c.8734A > G, p.(Arg2912Gly)] and one in the *BRIP1* [c.797C > T, p.(Thr266Met)] genes. All pipeline combinations detected both variants. The third patient carried an intronic VUS in the *ATM* gene [c.2838 + 10G > A]. This variant was detected only upon inclusion of interval padding. Likewise, the BWA Enrichment application failed to detect this intronic VUS.

### False positive calls

Bowtie2/GATK-UG analysis demonstrated the highest overall false positive rate (FPR) irrespective of interval padding (FPR = 59.16%) (Fig. 2a–c). This emerged due to frequent false positive SNV calls in exonic (FPR = 68.52%, Fig. 2i) and intronic regions (FPR = 62.5%, Fig. 2ii). Recurrent false positive intronic indels were detected in all GATK-HC pipelines (FPR = 52.22%, Fig. 2iv). Statistical analysis (Kruskal–Wallis test) demonstrated that variant calling, affects the rates of false positive intronic SNV calls in a statistically significant manner (Additional file 8). In detail, false positive intronic SNVs are not detected with GATK-HC (FPR = 0) compared to GATK-UG (FPR = 26.25%*,* *p* value = 0.011) and BWA Enrichment (FPR = 12.5%, *p* value = 0.046). However, in regards of intronic indels, GATK-HC exhibited an increased FPR (FPR = 52.22%, *p* value = 0.00158) compared to GATK-UG, SAMtools and BWA Enrichment (Fig. 2iv). Statistically significant FPR variances are shown in Additional file 8. At this point we need to note that a large proportion of false positive calls were detected in the *PMS2*_exon15*,* *PMS2*_exon7, *MSH2*_exon5, *MSH6*_exon1, *STK11*_exon3*,* *STK11*_exon9, *PTEN*_exon4*,* *PTEN*_exon3, *NF1*_exon1 and *NF1*_exon5 regions.

## Discussion

In this study we carried out a comprehensive comparison of the performance of short-read sequence alignment (BWA-MEM, Bowtie2, Stampy) and variant calling algorithms (GATK-HC, GATK-UG, SAMtools), in combination with interval padding length (null, 50 bp and 100 bp), for the analysis of targeted NGS data. Using targeted short-read data of 14 samples from a single NGS panel study of BC patients, we evaluated different pipelines based on several criteria, including mapping efficiency, depth of coverage, variant calling performance, detection of actionable variants and false positive rates. These results provide valuable information about the performance of the selected tools towards the molecular diagnosis of BC susceptibility, as well as insights for the selection of the most accurate variant calling pipeline, towards targeted-panel and exome sequencing data analysis.

Data pre-processing and variant discovery were performed according to EuroGentest and European Society of Human Genetics guidelines for the evaluation and validation of NGS applications, for the diagnosis of genetic disorders [24]. Although it was suggested that removal of duplicates has a minimal effect on variant calling accuracies [25], there exists a well-established recognition that removing duplicate reads cannot decrease the accuracy of variant calling. Hence, duplicates’ removal is regularly implemented, to limit any potential bias towards variant calling [20]. It is also well described that read trimming [26], indel realignment and base recalibration, increase the accuracy of variant calling [20, 22, 27]. Therefore, we implemented these steps as standard practice.

At first, we investigated mapping efficiencies for the BWA-MEM, Bowtie2 and Stampy aligners. Although, BWA-MEM and Bowtie2 demonstrated highly comparable mapping efficiencies, implementation of BWA-MEM mapping possessed the highest mapping efficiency. Our results agree with studies reporting that BWA-MEM possessed a lower number of misaligned reads compared to Bowtie2 [6, 9, 28]. Opposed to results reported by others [29], Stampy demonstrated the lowest mapping efficiency with over than 5% of unmapped reads. In addition, similar to a study by Cornish and Guda [29], despite the comparably higher mapping efficiency demonstrated by BWA-MEM, Bowtie2 achieved higher on average DP compared to BWA-MEM. Our results, confirm that tools which utilize similar algorithms may achieve similar results to each other [13, 18], since both the BWT-based algorithms (BWA-MEM, Bowtie2) achieved similar mapping efficiencies and outperformed Stampy (hash-based algorithm). However, we need to note that the difference in mapping efficiencies observed between the three alignment algorithms is relatively small. Hence, the read depth alone is unlikely to be a factor in the variant calling accuracy.

Herein, we present that the alignment method affects the total variant and exonic SNV calling performance (*p* value < 0.05) with Bowtie2 exhibiting reduced performance compared to BWA-MEM and Stampy. The top performing tier pipelines based on our comparisons are BWA-MEM/SAMtools with 100 bp padding, followed by BWA-MEM/SAMtools and Stampy/SAMtools pipelines with 50 bp padding, Stampy/SAMtools with 100 bp padding and BWA-MEM/GATK-UG with 50 bp padding. Likewise, Stampy/SAMtools with 50 bp padding followed by BWA-MEM/GATK-UG with zero and 50 bp padding, BWA-MEM/SAMtools with 100 bp padding and BWA Enrichment application, were the top tier exonic SNV calling pipeline combinations. Our results, partly agree with previous data [3, 4], supporting the finding that BWA-MEM/SAMtools pipeline showed the best performance for SNP calls. In contrast to what we present, Whang et al. [3] showed that the variant caller has more influence than read aligner on SNP calling, whereas Kumaran et al. [4] did not observe any significant changes in the top performing SNP calling pipelines. It is noteworthy, that other studies [6, 9] demonstrated that BWA-MEM consistently performed better than Bowtie2. Even so, precision and recall metrics varied greatly depending on the variant caller used, with GATK-UG being the best variant caller (for SNVs) irrespective of the alignment method used [6, 29]. This was also observed in our study, where BWA-MEM in combination with GATK-UG with null padding and 50 bp interval padding, detected all actionable exonic SNVs and accomplished high SNV calling performance. In agreement with this, other studies have shown that GATK-UG is better in calling coding SNVs compared to GATK-HC [30, 31] and SAMtools [8]. However, other studies demonstrated that GATK-HC [22], or SAMtools possess higher variant calling efficiencies compared to GATK-UG [9].

The precise detection of indels and intronic variants is more challenging since there are limited guidelines. It is interesting that in our hands, irrespective of interval padding and alignment algorithm, all GATK-UG based pipelines failed to detect indels—including truncating PVs—, compared to GATK-HC, SAMtools and BWA Enrichment. These results match the current knowledge that GATK-HC and SAMtools have a superior ability of calling indels, compared to GATK-UG [8, 11, 30, 32]. In addition, studies have shown that GATK-HC outperforms SAMtools with regards to indel calling [3, 4, 29, 32, 33], a result which agrees with our observations, since SAMtools based pipelines, failed to detect 50% of the intronic indels. The algorithms underlying HaplotypeCaller, SAMtools and UnifiedGenotyper also support this observation, since local de novo assembly methods used by HaplotypeCaller are more efficient around indel regions, compared to Bayesian calling methods [10]. Despite the higher indel recall rates demonstrated by GATK-HC, precision remained at low levels due to a high number of false positive indel calls. Hence, SAMtools demonstrated higher intronic indel calling efficiencies compared to GATK-HC and GATK-UG (*p* value < 0.05). Nevertheless, there are still reports supporting that indel calling efficiencies are better for the GATK-UG than GATK-HC [6, 10, 30, 31] or SAMtools [6, 10, 34]. In addition, we highlight that null interval padding and BWA Enrichment analysis, result in low intronic variant calling efficiencies and decreased detection rates of actionable PVs, including spliceogenic SNVs, since as expected interval padding highly affects variant calling in exon flanking regions. It is noted that while the GATK suite recommends interval padding in its forum, a portion of variant calling algorithms do not include options on this parameter and even if included, those are not required arguments. Thus, several users applying tools with nearly default parameters, may not be aware of the importance of interval padding for the analysis of their sequencing data. Hence, we are pointing out the significance of interval padding and suggest its adjustment to a required rather than an optional parameter.

While the sensitivity of each pipeline needs to remain at high levels, there is a great need to reduce the number of false positive variant calls. Bowtie2 in combination with GATK-UG calling, demonstrated overall, the highest false positive rate, irrespective of interval padding and variant type. This is due to the fact that it exhibited the highest false positive SNV calling rate. In addition to this, there is evidence that GATK-HC produces a large number of novel indels [30, 31]. Arguably, this corresponds to its high false positive indel calling rate compared to GATK-UG and SAMtools [30, 31]. We indeed noticed that GATK-HC exhibited a higher false positive indel calling rate within intronic regions, when interval padding (50 bp or 100 bp) was included in the pipeline. It is frequently observed that false positive calls are annotated as novel PVs [33] and located in genes being associated with the disease of interest. These calls usually appear when pseudogenes interfere with the variant calling process. In this report, the vast majority of false positive calls occured in the *PTEN*, *PMS2* and *NF1* genes which are known to bear pseudogenes that potentially affect the downstream analysis [35, 36]. We thus suggest that the validation of PVs using Sanger Sequencing is an important and decisive step.

The Genome Analysis Toolkit has been widely accepted and is regarded as the “Gold Standard”, especially for germline Illumina sequencing data [37]. It is constantly evolving with a diversity of performance optimization parameters [34]. Although a large number of studies pointed out that its variant callers present the best performance [8, 19, 20, 22, 37, 38], there is evidence that other variant calling algorithms such as CASAVA [39] and Scalpel [40], may outperform GATK when calling SNVs and indels. The GATK team mentions that GATK-HC and GATK-UG present an equal power of calling SNVs, however GATK-HC has a superior ability of calling indels (https://gatk.broadinstitute.org/). So as of GATK version 3.3, they recommend using GATK-HC in all cases, with no exceptions [41].

As discussed, seven out of fourteen samples carried PVs in established BC susceptibility genes. Among these, two splice site variants in three samples, were only detected upon inclusion of interval padding. Likewise, using the BWA Enrichment algorithm we failed to detect these PVs. Three additional samples carried frameshift PVs in the *BRCA1*, *BRCA2* and *PALB2* genes, which were detected by all pipeline combinations except those based on GATK-UG calling, irrespective of interval padding. In addition, one patient carried a missense *TP53* PV. Although all pipeline combinations achieved to detect the missense PV, this call was filtered out, subsequent to the VAF threshold except for three GATK-UG based pipeline combinations that managed to detect it with adequate VAF. However, we cannot ignore that this observation may be attributable to a possibility that this missense PV could be a true mosaic event with a low VAF (< 30%) and not an argument over which variant calling algorithms perform better [42]. Hence, we estimate that a large proportion of PVs will be missed when using pipelines with low precision and recall rates. As shown, these low detection rates can have direct clinical impact on patient management, since individuals carrying PVs can benefit from risk management strategies including closer surveillance at an earlier age, prophylactic surgery and chemoprevention, as well as more personalized targeted therapies.

Even though all twenty-eight pipeline combinations converge on a relatively large proportion of variants detected, there still exists a significant degree of variability, with near-default parameters. This discordance is a consequence of different alignment and variant calling methods, as well as the use of different alignment and variant calling parameters. It is important to note that the performance of the above-mentioned tools is by no means constant since they are continuing to improve over time, whereas algorithms which are only commercially available (such as NovoAlign [18]) were not assessed during this work. In addition, our findings focus only on germline targeted sequencing data.

We finally support the necessity of improving existing tools or developing new algorithms to achieve more reliable and more consistent calling results. Although our findings should be validated using a larger dataset, as well as explored further using different NGS panels, the outcome of our study has important implications for the diagnosis of BC susceptibility through panel testing in diagnostic molecular genetic testing laboratories, where the high quality of the offered clinical genetic tests is of paramount importance.

## Conclusions

We recommend the inclusion of interval padding and alignment with BWA-MEM for the accurate detection of intronic variants including spliceogenic PVs associated with the disease of interest. We also demonstrate that using nearly default parameters, the BWA Enrichment® failed to detect all the spliceogenic PVs and a missense PV in the *TP53* gene. We additionally suggest that GATK-HC and SAMtools should be used in combination for the accurate detection of indels, since GATK-HC demonstrates high recall rates, while SAMtools demonstrates high precision rates. Moreover, GATK-UG is suggested for the efficient detection of SNV calls. Finally, as precision medicine advances rapidly and NGS technologies are being widely integrated as a routine diagnostic tool, we highlight the necessity of accurate variant calling and bioinformatics expertise.

## Methods

### Sample selection

Results for the validation experiments described in this study were obtained from a targeted NGS-panel study of BC patients. Each study participant signed an informed consent form and agreed to undergo genetic testing. All study participants were carriers of known PVs or VUSs, which were identified previously by Sanger sequencing.

### Library preparation and sequencing

Library preparation was performed on genomic DNA samples using a panel of 94 cancer susceptibility genes (Illumina TruSight Cancer Sequencing panel—#FC-121-0202). The panel contains oligos targeting and enriching more than 1700 exons including coding regions and noncoding exon-flanking regions (~ 50 bp) spanning 94 cancer susceptibility genes (Additional file 1) [43]. The TruSight Rapid Capture kit was used for the library preparation according to the manufacturer’s protocol (Illumina, #FC-140-1106). Paired-end sequencing was performed on the NextSeq 500 Sequencing Platform (Illumina) using a High-Output v2.5 kit. We carried out two independent runs of 2 × 75 cycles and 2 × 150 cycles, aiming to examine the effect of read depth on subsequent variant calls.

### Data processing

**Fig. 5:**
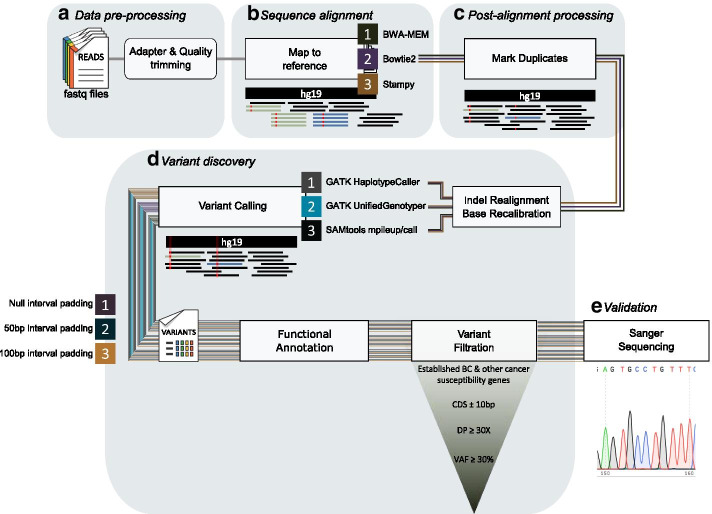
Data pre-processing, sequence alignment, post-alignment processing, variant discovery and validation workflow. Prior to sequence alignment, adapter and low-quality trimming were applied on the FASTQ files using the Cutadapt tool. Fastq files were then aligned to the hg19 reference human genome assembly (GRCh37) using the Burrows Wheeler Aligner (BWA)-Maximal Exact Match (MEM), Bowtie2 and Stampy sequence alignment algorithms. Following sequence alignment, sam files were sorted by coordinate using Picard SortSam tool. Duplicates were marked and removed using Picard MarkDuplicates tool and read groups were added using Picard AddOrReplaceReadGroups. Local realignment around indels (insertions/deletions) was performed using the Genome Analysis ToolKit (GATK) IndelRealigner tool and base quality score recalibration was performed using the GATK BaseRecalibrator tool. The GATK-UnifiedGenotyper, GATK-HaplotypeCaller and SAMtools mpileup/call algorithms were used for variant calling. Genetic variants were functionally annotated using the ANNOVAR tool. The workflow was repeated three times using the TruSight Cancer genomic interval file, with null, 50 bp and 100 bp interval padding. Data analysis was also performed using the Illumina’s BWA Enrichment application (not shown in the figure). *BC* breast cancer, *CDS* coding sequence, *DP* depth of coverage, *GATK* Genome Analysis ToolKit, *Indel* insertions/deletions, *VAF* variant allele frequency, *VUS* variant of uncertain clinical significance

In order to comply with international guidelines, data pre-processing and variant discovery were performed according to EuroGentest and European Society of Human Genetics recommendations for the evaluation and validation of NGS applications for the diagnosis of genetic disorders (Fig. 5, Additional file 2) [24].

Prior to mapping, adapter and low-quality trimming was performed on the FASTQ files, using the Cutadapt tool (v1.9) [44]. According to the Broad Institute recommendations, sequence reads were aligned to the hg19 reference human genome assembly (GRCh37, including decoy contigs) using the BWA-MEM algorithm (v0.7.17) [13], Bowtie2 (v2.3.5.1) [15] and Stampy sequence alignment algorithms (v1.0.32) [14]. Following mapping, SAM files were sorted by coordinate using the Picard (v2.20.3) (https://broadinstitute.github.io/picard/) SortSam tool. Duplicates were detected and removed using the Picard MarkDuplicates tool and read groups were added using the Picard AddOrReplaceReadGroups tool. Local realignment around indels was performed using the GATK (v3.6-0) (https://gatk.broadinstitute.org/hc/en-us) IndelRealigner tool and bases were recalibrated according to the best practice guidelines (GATK BaseRecalibrator). Depth of coverage was calculated using the GATK DepthOfCoverage tool. An interval file with the coordinates of the genomic regions targeted by the panel was downloaded from Illumina’s repository (https://support.illumina.com/downloads/nextera-flex-for-enrichment-enrichment-manifest-files.html) and used for variant calling. The interval file was used as such (null interval padding), or extended with 50 bp or 100 bp padding. Variant calling was performed using the GATK-UG, GATK-HC and SAMtools (v1.9) (http://samtools.github.io/bcftools/bcftools.html) mpileup and call tools. Alignment and variant calling were also performed using the BWA Enrichment (v2.1.2) application of Illumina, Inc. (https://basespace.illumina.com/apps/4797793, last accessed 27/01/2020), which includes BWA mapping and GATK variant calling. Adapter trimming was selected as an advanced option. Since 50 bp or 100 bp interval padding was not an option, 150 bp interval padding was included in the enrichment analysis.

Genetic variants were functionally annotated using ANNOVAR [45]. For our intended clinical validation purposes, variant calling assessment was only performed for established and clinically actionable BC predisposition genes (*BRCA1,* *BRCA2,* *PALB2,* *RAD51D,* *ATM,* *CHEK2,* *PTEN,* *TP53*) and other cancer predisposition genes (*CDH1,* *BRIP1,* *CDKN2A,* *MSH2,* *MSH6,* *NBN,* *NF1,* *PMS2,* *RAD51C,* *STK11*). Downstream analyses included variant filtration based on position (coding sequence ± 10 bp flanking regions), DP ≥ 30X and VAF ≥ 30%. Possible mosaic events (VAF < 30%) were excluded from the analysis. Twenty-eight variant calling pipeline combinations were compared, including combinations of sequence alignment, variant calling algorithms and interval padding lengths, along with Illumina’s BWA Enrichment application (Fig. 5). Detailed commands and parameters used are supplied in Additional file 9.

### Pipeline performance

For the pipeline performance evaluation, variants were categorized in four groups; exonic SNVs, exonic indels, intronic SNVs (± 1– ± 10) and intronic indels (± 1- ± 10). We verified all variants passing quality control filters by Sanger Sequencing. In the sequel, we defined true positive (TP), false positive (FP), true negative (TN) and false negative (FN) variants. True Positives are variant sites confirmed by Sanger Sequencing. True Negatives are sites correctly called as reference (sites were considered as true negatives if variants miscalled by other pipelines were not detected by the pipeline under investigation). False positives are reference sites miscalled as variants (not confirmed by Sanger Sequencing) and false negatives are variant sites, miscalled as reference.

Pipelines were ranked in the receiver operating characteristic (ROC) space. False positive and true positive rates (FPR and TPR) were calculated for the entity of variant calls and plotted on the ROC space. In the ROC space, each point corresponds to an instance of a confusion matrix (the 2 × 2 table that reports the number of FP, FN, TP and TN calls). The pipelines were ranked based on the perpendicular distance (*d*) of each point from the diagonal—“Random Guess” line. To further assess the pipeline’s performance, confusion matrices were analysed using the MCC, p, r and F1 metrics [46], which were calculated as shown below:

$$\begin{aligned}&Matthews\, Correlation\, Coefficient=MCC=\frac{TP\times TN-FP\times FN }{\sqrt{\left(TP+FP\right)\left(TP+FN\right)(TN+FP)(TN+FN)}}\\ &Precision=p =\frac{TP}{TP+FP}\\ &Recall= r=\frac{TP}{TP+FN}\\ &{F}_{1}\text{-}score=2 \times \frac{r \times p}{r+p}\end{aligned}$$

The d, MCC, p, r and F1 values were used to perform hierarchical clustering analysis based on the Lance–Williams agglomerative hierarchical clustering algorithm, which at each stage recomputes dissimilarities between clusters. The variant calling concordance of the top tier pipelines was analysed using Venn diagrams (http://bioinformatics.psb.ugent.be/webtools/Venn/).

It is worth noting that in contrast to SNVs, the genomic position of indels detected using NGS data analysis, is not always defined by a single, unambiguous coordinate [47]. In detail, the same insertion after position “i” (position in the gold standard data -Sanger Sequencing), can be also annotated as an insertion after positions i + 1 or i + 2 etc. These annotations have matching biological meaning and an identical position when validated by Sanger Sequencing. Thus, a clear NGS annotation of these variants should include all the alternative indel positions [47]. Consequently, although when comparing indels called by different algorithms, we treat them as TP if they are within the range of i ± 5 positions [32].

### Statistical analysis

The R (v3.3.2) (https://www.r-project.org/) statistical computing language was used for the statistical analyses presented in this manuscript. The Shapiro–Wilk’s and Levene’s tests were used to test normality and equality of variances for variables calculated for three or more groups. The one-way ANOVA parametric test was used to compare the means of homogeneous, normally distributed and independent numerical variables. The non-parametric Kruskal–Wallis test was used to compare numerical variables of three or more groups when one-way ANOVA assumptions (homogeneity and normality of variances) were not met. Post-hoc multiple comparisons were performed using the Bonferroni method [48]. A *p* value of less than 0.05 was considered to be statistically significant.

## Supplementary Information

**Additional file 1: Table S1.** TruSight Cancer (Illumina) target genes in alphabetical order.

**Additional file 2: Table S2.** EuroGentest and the European Society of Human Genetics, guidelines for the evaluation and validation of NGS applications.

**Additional file 3: Figure S1.** Sequencing and Mapping Evaluation. **a**. Number of reads and clusters generated per run. **b** Number of unmapped reads per sample and alignment method.

**Additional file 4: Table S3.** Number of reads per sample and mapping tool.

**Additional file 5: Table S4.** Minimum, mean and maximum depth of coverage, per gene and alignment algorithm. Depth of coverage results upon NextSeq 500 High-Output kit 2x75 cycles and 2x150 cycles sequencing.

**Additional file 6: Figure S2.** Hierarchical clustering of the tools. The d, MCC, p, r and F1 values were used to perform hierarchical clustering analysis based on the Lance–Williams agglomerative hierarchical clustering algorithm, which at each stage recomputes dissimilarities between clusters.

**Additional file 7: Figure S3.** Box plot of Matthew Correlation coefficient (MCC) comparisons per alignment, variant calling or padding method. Each dot represents one observation and horizontal bold lines denote median MCC values. Boxes extend from the 25th to the 75th percentile of each group’s distribution of values. Vertical extending lines (whiskers) denote the upper and lower adjacent values. Statistical analyses were performed using the non-parametric Kruskal-Wallis or one-way ANOVA tests.

**Additional file 8: Figure S4.** Box plot comparisons of False Positive Rates (FPR). Only statistically significant differences are shown. Each dot represents one observation and horizontal bold lines denote median FPR values. Boxes extend from the 25th to the 75th percentile of each group’s distribution of values. Vertical extending lines (whiskers) denote the upper and lower adjacent values. Statistical analysis was performed using the non-parametric Kruskal-–Wallis test.

**Additional file 9.** Detailed commands and parameters used for data pre-processing, sequence alignment, post-alignment processing and variant discovery.

## Data Availability

All summary data included in results are included in supplementary material. The data and code underlying this article are readily available upon request.

## Abbreviations

BC: Breast cancer
BWA: Burrows–Wheeler Aligner
BWT: Burrows–Wheeler transform
CDS: Coding sequence
*d*: Distance
DP: Depth of coverage
FN: False negative
FP: False positive
FPR: False positive rate
GATK: Genome analysis ToolKit
HC: HaplotypeCaller
indel: Insertion/deletion
MCC: Matthews correlation coefficient
MEM: Maximal Exact Match
NGS: Next-generation sequencing
p: Precision
PV: Pathogenic variant
r: Recall
ROC: Receiver operating characteristic
SNV: Single nucleotide variant
TN: True negative
TP: True positive
TPR: True positive rate
UG: UnifiedGenotyper
VAF: Variant allele frequency
VUS: Variant of uncertain clinical significance
