# Supplementary material for: Performance evaluation of pipelines for mapping, variant calling and interval padding, for the analysis of NGS germline panels
Source: BMC Bioinformatics. 2021 Apr 28;22:218. doi: 10.1186/s12859-021-04144-1 (PMC8080428; doi:10.1186/s12859-021-04144-1)
Supplement: Supplementary file 1 — Additional file 1: Table S1. TruSight Cancer (Illumina) target genes in alphabetical order. [file 12859_2021_4144_MOESM1_ESM.pdf]

Supplementary Table 1: TruSight Cancer (Illumina) target genes in alphabetical order

AIP	*ALK*	*APC*	*ATM*	*BAP1*	*BLM*	*BMPR1A*	*BRCA1*	*BRCA2*	*BRIP1*
BUB1B	*CDC73*	*CDH1*	*CDK4*	*CDKN1C*	*CDKN2A*	*CEBPA*	*CEP57*	*CHEK2*	*CYLD*
DDB2	*DICER1*	*DIS3L2*	*EGFR*	*EPCAM*	*ERCC2*	*ERCC3*	*ERCC4*	*ERCC5*	*EXT1*
EXT2	*EZH2*	*FANCA*	*FANCB*	*FANCC*	*FANCD2*	*FANCE*	*FANCF*	*FANCG*	*FANCI*
FANCL	*FANCM*	*FH*	*FLCN*	*GATA2*	*GPC3*	*HNF1A*	*HRAS*	*KIT*	*MAX*
MEN1	*MET*	*MLH1*	*MSH2*	*MSH6*	*MUTYH*	*NBN*	*NF1*	*NF2*	*NSD1*
PALB2	*PHOX2B*	*PMS1*	*PMS2*	*PRF1*	*PRKAR1A*	*PTCH1*	*PTEN*	*RAD51C*	*RAD51D*
RB1	*RECQL4*	*RET*	*RHBDF2*	*RUNX1*	*SBDS*	*SDHAF2*	*SDHB*	*SDHC*	*SDHD*
SLX4	*SMAD4*	*SMARCB1*	*STK11*	*SUFU*	*TMEM127*	*TP53*	*TSC1*	*TSC2*	*VHL*
WRN	*WT1*	*XPA*	*XPC*						
