# Supplementary material for: Performance evaluation of pipelines for mapping, variant calling and interval padding, for the analysis of NGS germline panels
Source: BMC Bioinformatics. 2021 Apr 28;22:218. doi: 10.1186/s12859-021-04144-1 (PMC8080428; doi:10.1186/s12859-021-04144-1)
Supplement: Supplementary file 2 — Additional file 2: Table S2. EuroGentest and the European Society of Human Genetics, guidelines for the evaluation and validation of NGS applications. [file 12859_2021_4144_MOESM2_ESM.pdf]

Supplementary Table 2: The EuroGentest and the European Society of Human Genetics guidelines for the evaluation and validation of NGS applications

Processing step	Description	Tools and databases	Output
**Primer trimming**	In amplicon sequencing primers have to be trimmed from the reads	CutAdapt, BWA (soft clipping while mapping)	FASTQ files
**Adapter trimming (optional)**	Sequencing adapters may be trimmed from the read ends for those reads where the insert size is smaller than the read length.	CutAdapt, BWA (soft clipping while mapping), Trimmomatic, SeqPrep	FASTQ files
**Low-quality trimming (optional)**	Low quality bases may also interfere with mapping and variant calling and can be trimmed from the end (and begin) of reads.	CutAdapt, BWA (soft clipping while mapping), Trimmomatic, SeqPrep	FASTQ files
**Mapping**	In the read mapping step, paired-end/ single-end reads are mapped to the reference genome allowing for base changes and indels. Mapping should always be performed against the full reference genome even when a small gene panel is sequenced.	BWA, Novalign, Stampy, SOAP2, Bowtie	BAM file
**Duplicate removal (optional)**	In shotgun sequencing few duplicates are expected since the DNA is randomly sheared. However, duplicates can occur during PCR and as an artifact of imaging. In amplicon sequencing, duplicates are expected and should not be removed.	Picard MarkDuplicates	BAM file
**Indel realignment (optional)**	The presence of indels in the sequenced samples often leads to multiple single base mismatches around these sites, especially if they reside close to the start or end of reads. These artifacts may show up as false-positive variants during subsequent analysis. Local re-alignment algorithms identify such positions and try to minimize the amount of mismatching bases by performing a local re-alignment of the indel spanning reads, increasing the accuracy of the calls while minimizing false positives.	GATK RealignerTargetCreator & IndelRealigner and SRMA	BAM file
**Quality score recalibration (optional)**	After mapping to the reference genome, the base quality score of the reads can be recalibrated to better match the probability of false base calls and to spread the quality scores wider over the valid range. In most algorithms, false base calls are distinguished from real variants by performing a simple base calling or using databases of known polymorphisms.	GATK BaseRecalibrator & PrintReads, ReQON	BAM file
**Variant calling**	Variant calling consists of detecting and genotyping differences to the reference genome (base changes and small indels).	GATK UnifiedGenotyper, GATK HaplotypeCaller, samtools and Platypus	VCF file
**Annotation**	Variant interpretation requires detailed annotation. Very basic annotations are gene name, region (exonic, splicing, intronic, intergenic, etc.) and coding change information. Additionally, minor allele frequency for known polymorphisms, pathogenicity and conservation scores and clinical databases can be used.	Annovar, SNPeff, Cartagenia Bench Lab NGS, dbSNP, 1000 Genomes, ESP 6500, SIFT, PhyloP, MutationTaster, COSMIC, OMIM, ClinVar, HGMD	CSV, TSV, TXT, excel files or database

In red, algorithms used in this study.
