# Supplementary material for: Performance evaluation of pipelines for mapping, variant calling and interval padding, for the analysis of NGS germline panels
Source: BMC Bioinformatics. 2021 Apr 28;22:218. doi: 10.1186/s12859-021-04144-1 (PMC8080428; doi:10.1186/s12859-021-04144-1)

Supplementary Figure 1. Sequencing and Mapping Evaluation.

A. Number of reads and clusters generated per run.

B. Number of unmapped reads per sample and alignment method.

A

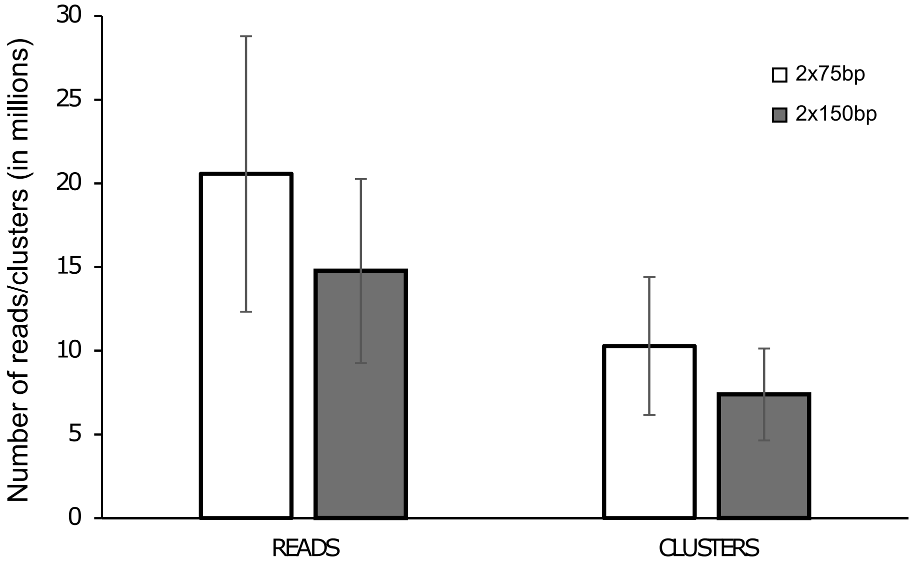

Reads	
**2x75bp**	**2x150bp**
20,563,478	14,768,439
±8,229,493	±5,493,299
**Clusters**	
**2x75bp**	**2x150bp**
10,281,739	7,384,220
±4,114,747	±2,746,649

B

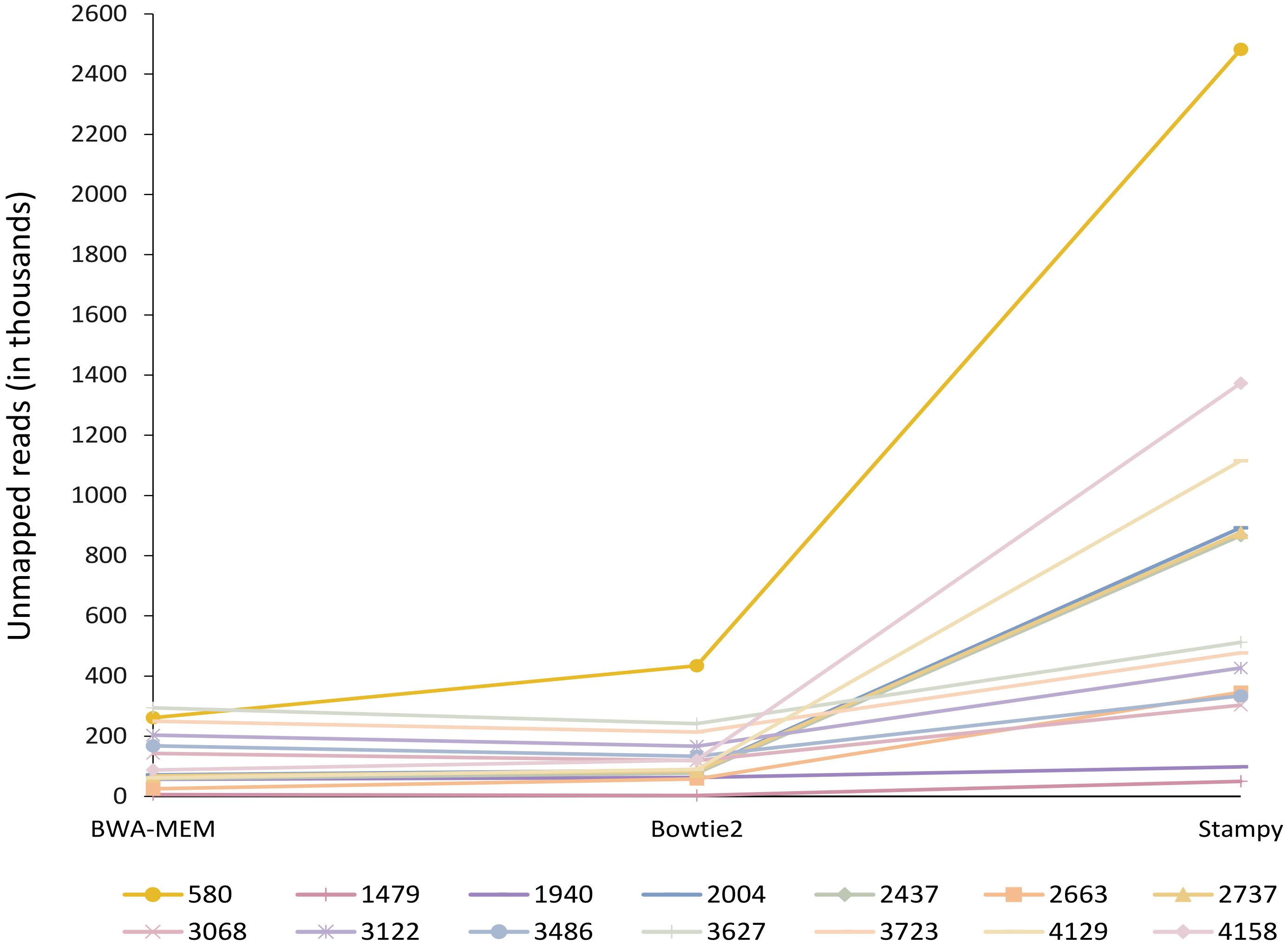

Supplement: Supplementary file 3 — Additional file 3: Figure S1. Sequencing and Mapping Evaluation. a. Number of reads and clusters generated per run. b Number of unmapped reads per sample and alignment method. [file 12859_2021_4144_MOESM3_ESM.pdf]
