# Supplementary material for: Performance evaluation of pipelines for mapping, variant calling and interval padding, for the analysis of NGS germline panels
Source: BMC Bioinformatics. 2021 Apr 28;22:218. doi: 10.1186/s12859-021-04144-1 (PMC8080428; doi:10.1186/s12859-021-04144-1)
Supplement: Supplementary file 4 — Additional file 4: Table S3. Number of reads per sample and mapping tool. [file 12859_2021_4144_MOESM4_ESM.pdf]

Supplementary Table 3: Number of reads per sample and mapping tool

Sample	BWA-MEM	Bowtie2	Stampy	BWA-MEM	Bowtie2	Stampy
	Unmapped reads			Mapped reads		
580	261414	433927	2482228	13577776	13393773	11345472
1479	5729	2986	49875	2732903	2735470	2688581
1940	56843	62598	98158	3778542	3772712	3737152
2004	71512	86887	892862	10914881	10890053	10084078
2437	58160	77503	866615	13543000	13508299	12719187
2663	25346	57861	346134	3815622	3774685	3486412
2737	68244	84019	877024	10206196	10180719	9387714
3068	142531	119111	303369	17504029	17527195	17342937
3122	203406	166814	426814	24484793	24521020	24261020
3486	168354	133304	334370	20074059	20108832	19907766
3627	294525	241683	511994	25950782	26003375	25733064
3723	250282	213840	477146	28946629	28982724	28719418
4129	61594	90145	1115345	12408854	12375569	11350369
4158	87572	120406	1373053	20544836	20480896	19228249
Mean ± SD (%)	0.81 ± 0.43	0.97 ± 0.70	5.62 ± 4.56	99.19 ± 0.43	99.03 + 0.70	94.38 ± 4.56
