# Supplementary material for: Performance evaluation of pipelines for mapping, variant calling and interval padding, for the analysis of NGS germline panels
Source: BMC Bioinformatics. 2021 Apr 28;22:218. doi: 10.1186/s12859-021-04144-1 (PMC8080428; doi:10.1186/s12859-021-04144-1)
Supplement: Supplementary file 5 — Additional file 5: Table S4. Minimum, mean and maximum depth of coverage, per gene and alignment algorithm. Depth of coverage results upon NextSeq 500 High-Output kit 2x75 cycles and 2x150 cycles sequencing. [file 12859_2021_4144_MOESM5_ESM.pdf]

Supplementary Table 4: Minimum, mean and maximum depth of coverage, per gene and alignment algorithm. Depth of coverage results upon NextSeq 500 High-Output kit 2x75 cycles and 2x150 cycles sequencing.

	2x75 cycles									2x150 cycles								
	BWA-MEM			Bowtie2			Stampy			BWA-MEM			Bowtie2			Stampy		
	Min	Mean	Max	Min	Mean	Max	Min	Mean	Max	Min	Mean	Max	Min	Mean	Max	Min	Mean	Max
BRCA1	41	387	938	41	386	937	41	386	937	91	766	1583	93	766	1583	85	734	1558
BRCA2	20	389	987	20	389	980	20	389	987	99	773	1876	98	773	1862	94	749	1835
PALB2	36	433	1042	36	432	1041	36	432	1041	89	820	1987	87	822	1994	77	787	1950
BRIP1	30	387	1057	30	386	1057	30	386	1058	115	740	1963	116	738	1967	111	716	1941
NF1	5	354	1077	4	355	1048	5	354	1084	49	643	2101	49	643	1965	29	616	2071
MSH2	2	387	936	11	387	934	4	386	937	25	778	2014	42	780	1969	16	748	1992
MSH6	7	353	952	8	353	952	5	352	952	21	717	2081	25	719	2017	19	678	1967
PMS2	32	458	1188	38	458	1201	30	439	1187	97	834	2454	96	849	2459	65	781	2425
CDKN2A	54	325	911	60	328	913	53	323	913	120	649	1384	124	651	1387	96	604	1285
RAD51C	42	405	988	43	403	988	43	404	988	128	801	1795	126	797	1794	122	773	1791
RAD51D	103	398	1056	99	396	1055	104	397	1055	208	808	1698	206	812	1706	185	761	1516
NBN	34	401	1165	34	400	1175	33	400	1164	94	771	1990	94	768	1986	91	748	1970
ATM	14	397	1155	14	396	1165	13	396	1154	107	804	2282	118	804	2283	89	775	2240
CHEK2	21	380	1007	21	381	1156	21	373	955	82	675	1528	84	682	1538	71	648	1508
TP53	23	321	1029	26	322	1035	23	319	1035	51	590	1571	52	594	1599	45	533	1426
PTEN	21	421	1055	21	427	1100	21	411	979	109	664	1522	125	699	1641	84	635	1498
STK11	19	335	1058	19	337	1058	19	334	1058	87	700	2145	86	706	2155	82	646	2014
CDH1	18	394	1043	18	394	1043	18	392	1043	55	692	1594	51	694	1599	46	647	1563
	2	385±35	1188	4	385±35	1201	4	382±33	1187	21	735±69	2454	25	739±68	2459	16	699±73	2425
