# Supplementary material for: Performance evaluation of pipelines for mapping, variant calling and interval padding, for the analysis of NGS germline panels
Source: BMC Bioinformatics. 2021 Apr 28;22:218. doi: 10.1186/s12859-021-04144-1 (PMC8080428; doi:10.1186/s12859-021-04144-1)
Supplement: Supplementary file 6 — Additional file 6: Figure S2. Hierarchical clustering of the tools. The d, MCC, p, r and F1 values were used to perform hierarchical clustering analysis based on the Lance–Williams agglomerative hierarchical clustering algorithm, which at each stage recomputes dissimilarities between clusters. [file 12859_2021_4144_MOESM6_ESM.pdf]

Supplementary Figure 2: Hierarchical clustering of the tools

The d, MCC, p, r and F1 values were used to perform hierarchical clustering analysis based on the Lance–Williams agglomerative hierarchical clustering algorithm, which at each stage recomputes dissimilarities between clusters.

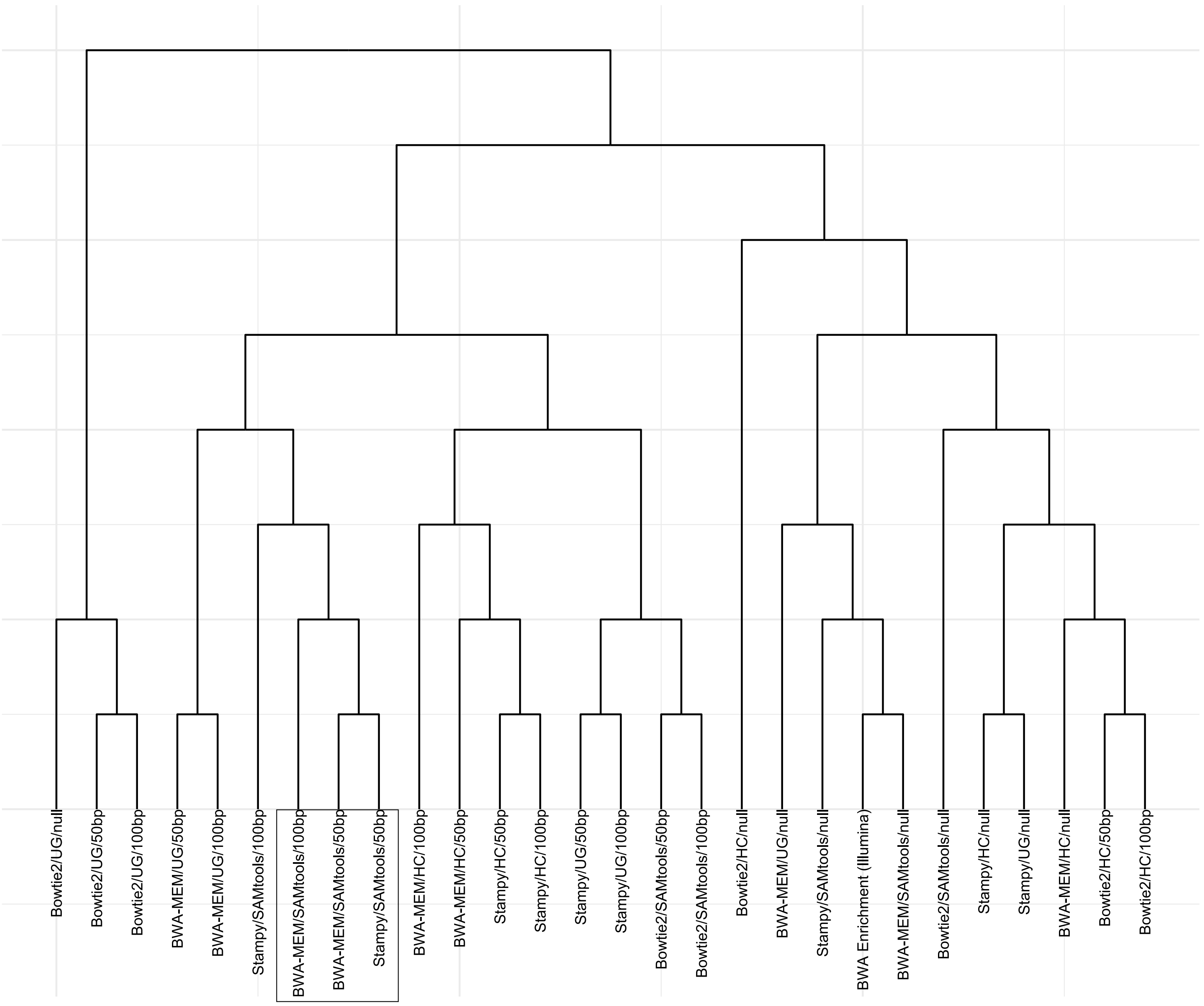

HC, GATK HaplotypeCaller; UG, GATK UnifiedGenotyper; null, zero interval padding; 50bp, 50bp interval padding; 100bp, 100bp interval padding. Denoted with box, top performing pipelines based on the perpendicular distance from the random-guess line.
