# Supplementary material for: Performance evaluation of pipelines for mapping, variant calling and interval padding, for the analysis of NGS germline panels
Source: BMC Bioinformatics. 2021 Apr 28;22:218. doi: 10.1186/s12859-021-04144-1 (PMC8080428; doi:10.1186/s12859-021-04144-1)

Supplementary Figure 3. Box plot of Matthews correlation coefficient (MCC) comparisons per alignment, variant calling or padding method. Each dot represents one observation and horizontal bold lines denote median MCC values. Boxes extend from the 25th to the 75th percentile of each group's distribution of values. Vertical extending lines (whiskers) denote the upper and lower adjacent values. Statistical analyses were performed using the non-parametric Kruskal-Wallis or one-way ANOVA tests.

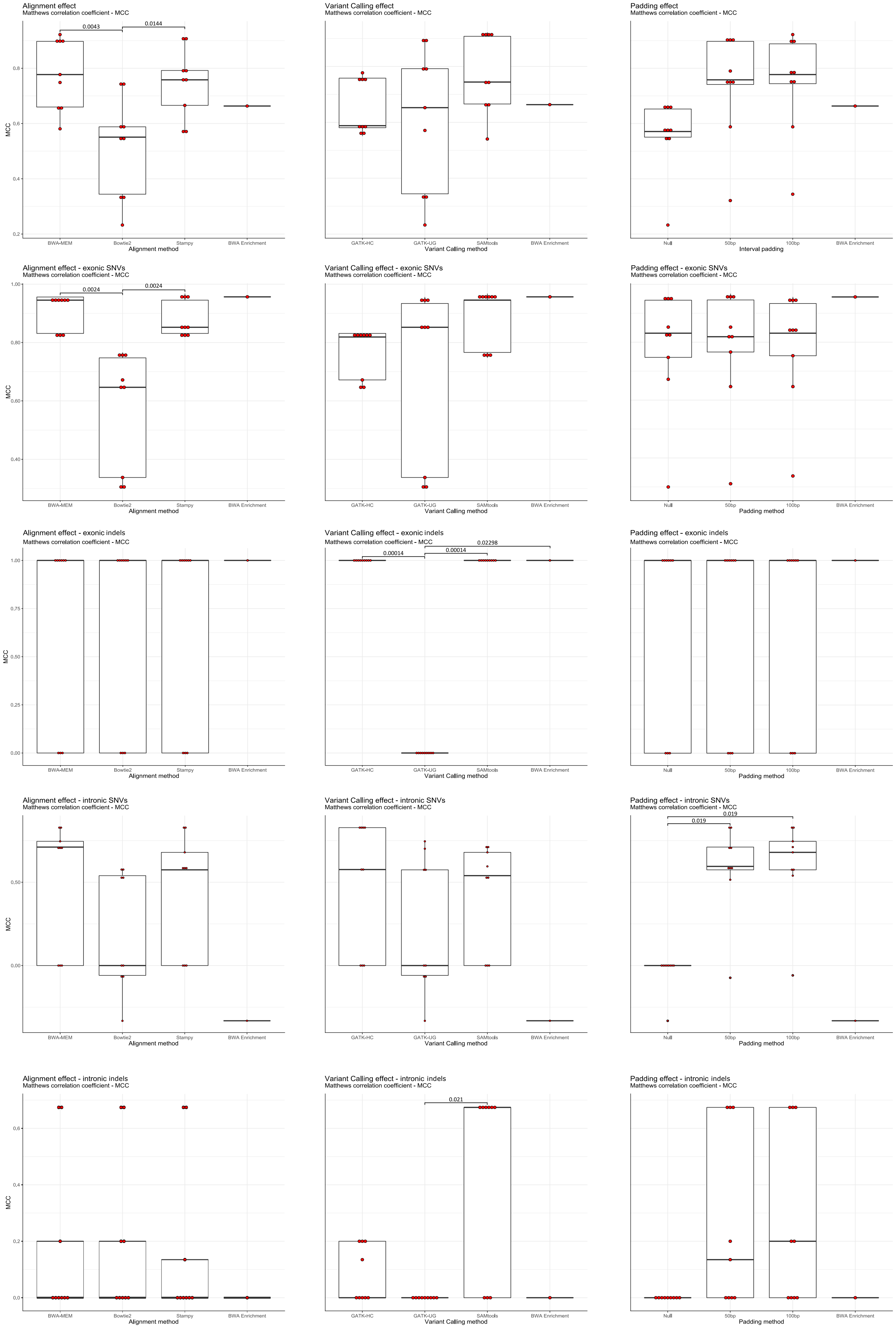

Supplement: Supplementary file 7 — Additional file 7: Figure S3. Box plot of Matthew Correlation coefficient (MCC) comparisons per alignment, variant calling or padding method. Each dot represents one observation and horizontal bold lines denote median MCC values. Boxes extend from the 25th to the 75th percentile of each group’s distribution of values. Vertical extending lines (whiskers) denote the upper and lower adjacent values. Statistical analyses were performed using the non-parametric Kruskal-Wallis or one-way ANOVA tests. [file 12859_2021_4144_MOESM7_ESM.pdf]
