# Supplementary material for: Performance evaluation of pipelines for mapping, variant calling and interval padding, for the analysis of NGS germline panels
Source: BMC Bioinformatics. 2021 Apr 28;22:218. doi: 10.1186/s12859-021-04144-1 (PMC8080428; doi:10.1186/s12859-021-04144-1)

Supplementary Figure 4. Box plot comparisons of False Positive Rates (FPR). Only statistically significant differences are shown. Each dot represents one observation and horizontal bold lines denote median FPR values. Boxes extend from the 25th to the 75th percentile of each group's distribution of values. Vertical extending lines (whiskers) denote the upper and lower adjacent values. Statistical analysis was performed using the non-parametric Kruskal-Wallis test.

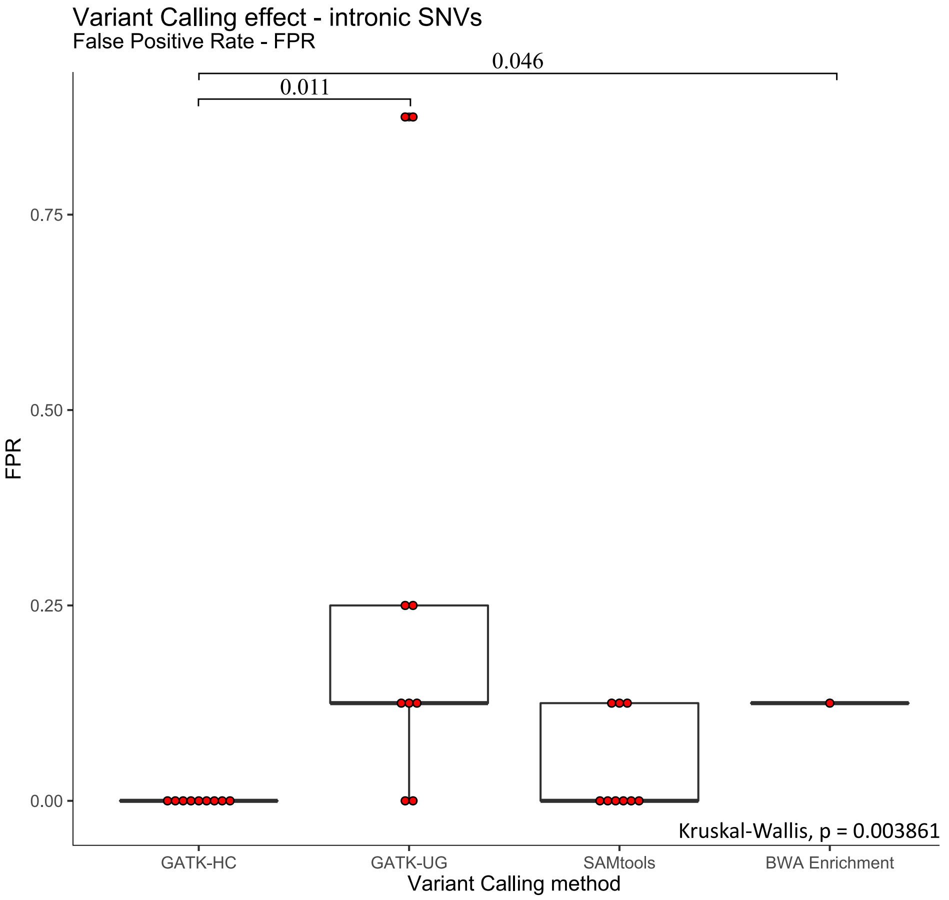

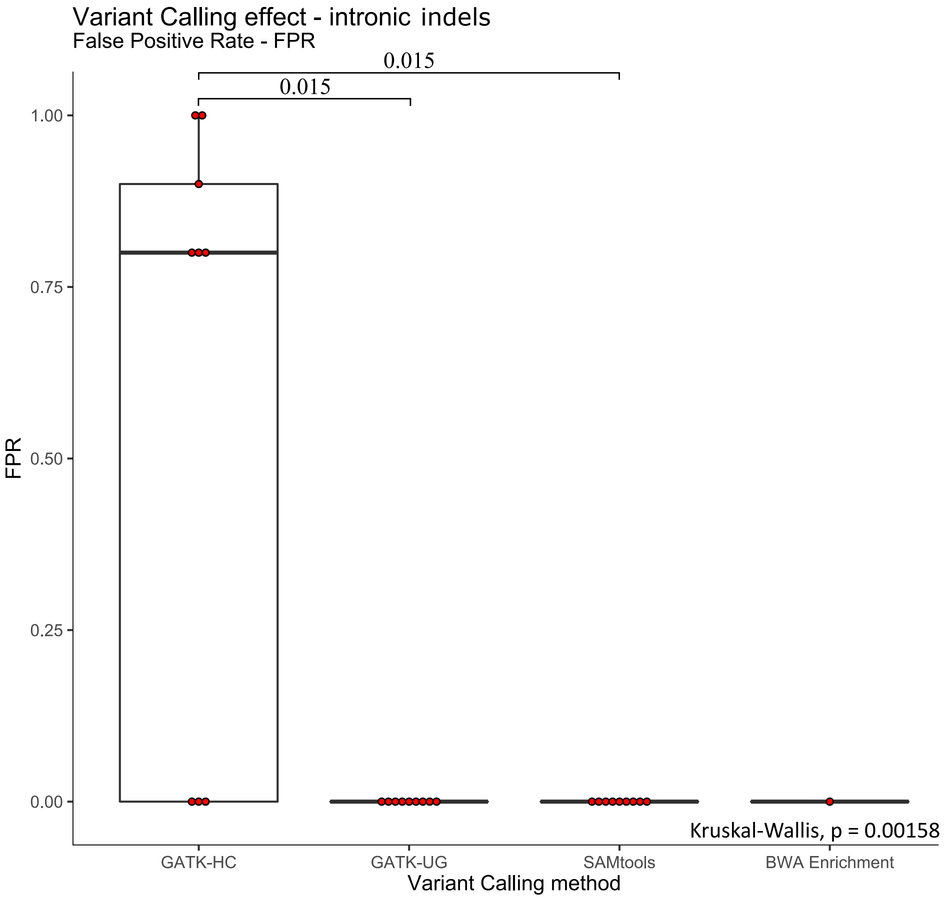

Supplement: Supplementary file 8 — Additional file 8: Figure S4. Box plot comparisons of False Positive Rates (FPR). Only statistically significant differences are shown. Each dot represents one observation and horizontal bold lines denote median FPR values. Boxes extend from the 25th to the 75th percentile of each group’s distribution of values. Vertical extending lines (whiskers) denote the upper and lower adjacent values. Statistical analysis was performed using the non-parametric Kruskal-–Wallis test. [file 12859_2021_4144_MOESM8_ESM.pdf]
