# Supplementary material for: Performance evaluation of pipelines for mapping, variant calling and interval padding, for the analysis of NGS germline panels
Source: BMC Bioinformatics. 2021 Apr 28;22:218. doi: 10.1186/s12859-021-04144-1 (PMC8080428; doi:10.1186/s12859-021-04144-1)
Supplement: Supplementary file 9 — Additional file 9. Detailed commands and parameters used for data pre-processing, sequence alignment, post-alignment processing and variant discovery. [file 12859_2021_4144_MOESM9_ESM.pdf]

Detailed commands and parameters used for data pre-processing, sequence alignment, post-alignment processing and variant discovery

Data pre-processing and variant discovery were performed according to EuroGentest and European Society of Human Genetics guidelines for the evaluation and validation of NGS applications for the diagnosis of genetic disorders.

Requirements

All third-party tools must be installed and correctly assigned to the respective variables defined within the files. The hg19 reference human genome assembly (GRCh37) must be installed and provided for alignment. Additional database files are required for filtering and annotation. Required inputs to all pipelines are paired FASTQ files of one or more samples. Paired FASTQ files are recognized based on the substring _R? - this should therefore be the only capital R contained in the FASTQ name and while additional characters may follow, different samples should be distinguished by whatever comes before.

DATA PRE-PROCESSING

Adapter & Quality Trimming

Prior to mapping, adapter and low-quality trimming was performed on the FASTQ files, using the Cutadapt tool (v1.9).

Commands and Options

```
trim_galore -q 20 --fastqc --length 80 --clip_R1 15 --clip_R2 15 --three_prime_clip_R1 10 --
three_prime_clip_R2 10 --stringency 5 --dont_gzip --no_report_file -o <output_directory> --paired
R1.fq R2.fq --path_to_cutadapt <path_to_cutadapt>

  •  -q/--quality <INT>
       Trim ends with less than <INT>Q-score (Phred Score) from reads
  •  --fastqc
       Run fastqc on the .fastq file once trimming is complete
  •  --length <INT>
       Discard reads shorter than the specified length
  •  --clip_R1 <INT>
       Remove the specified <INT>bp from the 5' end of read 1
  •  --clip_R2 <INT>
       Remove the specified <INT>bp from the 5' end of read 2
  •  --three_prime_clip_R1 <INT>
       Remove the specified <INT>bp from the 3' end of read 1
  •  --three_prime_clip_R2 <INT>
       Remove the specified <INT>bp from the 3' end of read 2
  •  --stringency <INT>
       overlap with adapter sequence required to trim a sequence
  •  --dont_gzip
       Output files won't be compressed with gzip
  •  --no_report_file
       No report file will be generated
  •  -o/--output_dir <DIR>
       output directory
  •  --paired
       Specified for paired-end files. Paired-end fastq files (R1.fq, R2.fq) follow the
       argument
  •  --path_to_cutadapt </path/to/cutadapt>
       Path to the CutAdapt executable
```

SEQUENCE ALIGNMENT

Sequence reads were aligned to the hg19 reference human genome assembly (GRCh37) using the Burrows Wheeler Aligner (BWA)-Maximal Exact Match (MEM) algorithm (v0.7.17), Bowtie2 (v2.3.5.1), and Stampy sequence alignment algorithms (v1.0.32).

Commands and Options

1. **BWA-MEM**

```
bwa mem hg19.fa -t 4 -M R1.fq R2.fq > output.sam

    •   -t <INT>
          Number of threads
    •   -M
          Mark shorter split hits as secondary (for Picard compatibility)
```

2. **Bowtie2**

```
bowtie2 -x <bt2-idx> --local -1 R1.fq -2 R2.fq -S output.sam

    •   -x <bt2-idx>
          The basename of the bowtie index for the reference genome
    •   --local
        Local Alignment. It does not require that the entire read align from one end to the
        other.
    •   -1 <fastq>
          Specified for forward read. R1 fastq file (R1.fq) follows the argument
    •   -2 <fastq>
          Specified for reverse read. R2 fastq file (R2.fq) follows the argument
    •   -S <sam>
          File to write SAM alignments to.
```

3. **Stampy**

```
bwa aln -q10 -t8 hg19.fa R1.fq > R1.sai
bwa aln -q10 -t8 hg19.fa R2.fq > R2.sai

    •   -q <INT>
          Parameter for read trimming.
    •   -t <INT>
          Number of threads

bwa sampe hg19.fa R1.sai R2.sai R1.fq R2.fq | samtools view -Sb - > output.bam

    •   -S
          Required for SAM input formats
    •   -b
          Required for BAM output formats

python stampy.py -g hg19.fa -h hg19.fa -t8 --bamkeepgoodreads -M output.bam

    •   -g <ref>
          Argument followed by the reference genome fasta file (hg19.fa)
    •   -h <ref>
          Argument followed by the reference genome fasta file (hg19.fa)
    •   -t <INT>
          Number of threads
    •   --bamkeepgoodreads
          Argument that copies the well-mapped reads to the output without remapping them
    •   -M <bam>
          Input bam file
```

POST-ALIGNMENT PROCESSING

Mark Duplicates

Following mapping, SAM files were sorted by coordinate using the Picard (v2.20.3) SortSam tool. Duplicates were detected and removed using the Picard MarkDuplicates tool and read groups were added using the Picard AddOrReplaceReadGroups tool.

Commands and Options

```
java -jar picard.jar MarkDuplicates \
INPUT=input.bam \
OUTPUT=marked_duplicates.bam \
REMOVE_DUPLICATES=true METRICS_FILE=metrics CREATE_INDEX=True \
VALIDATION_STRINGENCY=SILENT

    •   INPUT <bam>
          Argument followed by the input bam file
    •   OUTPUT <bam>
          Argument followed by the output bam file
    •   REMOVE_DUPLICATES <true/false>
          If true duplicates are removed from the output file. If false duplicates are
          flagged.
    •   METRICS_FILE <file>
          File to write duplication metrics
    •   CREATE_INDEX <true/false>
          Whether to create a BAM index when writing a coordinate-sorted BAM file
    •   VALIDATION_STRINGENCY <STRICT/SILENT>
          Setting to SILENT can improve performance when processing a BAM file with
          variable-length data (read, qualities, tags)
```

VARIANT DISCOVERY

Local realignment around INDELs (insertions/deletions) was performed using the Genome Analysis ToolKit (GATK) (v3.6-0) IndelRealigner tool and bases were recalibrated according to the best practice guidelines (GATK BaseRecalibrator). Depth of coverage was calculated using the GATK DepthOfCoverage tool. An interval file with the coordinates of the genomic regions targeted by the panel was downloaded from Illumina's repository and used for variant calling. The interval file was used as such (null interval padding), or extended with 50bp or 100bp padding. Variant calling was performed using the GATK-UnifiedGenotyper, GATK-HaplotypeCaller and SAMtools (v1.9) mpileup and call tools. Genetic variants were functionally annotated using ANNOVAR. Coverage evaluation was performed using the GATK DepthOfCoverage tool.

Indel Realignment

Commands and Options

```
java -jar GenomeAnalysisTK.jar -T RealignerTargetCreator \
-R hg19.fa \
-I duplicates.bam \
--known Mills_and_1000G_gold_standard.indels.hg19.sites.vcf \
-o sample.intervals

    •   -R <ref>
          Argument followed by the reference genome fasta file (hg19.fa)
    •   -I <bam>
          Argument followed by the input bam file
    •   --known <vcf>
          Argument followed by known reference indel sites in vcf format
    •   -o <intervals>
          Argument followed by the output file with .intervals extension (represents
          sites of extant and potential indels)

java -jar GenomeAnalysisTK.jar -T IndelRealigner \
-R hg19.fa \
-I duplicates.bam \
-known Mills_and_1000G_gold_standard.indels.hg19.sites.vcf \
-targetIntervals sample.intervals \
-o realigned.bam

    •   -R <ref>
          Argument followed by the reference genome fasta file (hg19.fa)
    •   -I <bam>
          Argument followed by the input bam file
    •   --known <vcf>
          Argument followed by known reference indel sites in vcf format
    •   -targetIntervals <intervals>
          Argument followed by the RealignerTargetCreator output file with .intervals
          Extension
    •   -o <bam>
          Argument followed by the bam output file
```

Base Recalibration

Commands and Options

```
java -jar GenomeAnalysisTK.jar -T BaseRecalibrator \
-R hg19.fa \
-knownSites dbsnp_138.hg19.vcf \
-knownSites 1000G_phase1.indels.hg19.sites.vcf \
-knownSites Mills_and_1000G_gold_standard.indels.hg19.sites.vcf \
-o recalibration_report.grp \
-I realigned.bam \
-L manifest.bed \
--interval-padding 50

    •   -R <ref>
          Argument followed by the reference genome fasta file (hg19.fa)
    •   --knownSites <vcf>
          Argument followed by vcf files of pne or more databases of known polymorphic
          sites used to exclude regions around known polymorphisms from analysis.
    •   -o <grp>
          Argument followed by the output recalibration table with .grp extension
    •   -I <bam>
          Argument followed by the input bam file
    •   -L <bed>
          Argument followed by the interval list file with .bed extension
    •   --interval-padding <INT>
          Amount of padding (in bp) to add to each interval.

java -jar GenomeAnalysisTK.jar -T PrintReads \
-R hg19.fa \
-BQSR recalibration_report.grp \
-o recalibrated.bam \
-I realigned.bam \
-L manifest.bed \
--interval-padding 50

    •   -R <ref>
          Argument followed by the reference genome fasta file (hg19.fa)
    •   -BQSR <grp>
          Argument followed by the recalibration table produced by BaseRecalibrator with
        .grp extension
    •   -o <bam>
          Argument followed by the bam output file
    •   -I <bam>
          Argument followed by the input bam file
    •   -L <bed>
          Argument followed by the interval list file with .bed extension
    •   --interval-padding <INT>
          Amount of padding (in bp) to add to each interval.
```

Variant Calling

Commands and Options

1. GATK HaplotypeCaller

```
java -jar GenomeAnalysisTK.jar -T HaplotypeCaller \
-R hg19.fa \
-I recalibrated.bam \
-dt none \
--emit-ref-confidence GVCF \
--variant_index_type LINEAR \
--variant_index_parameter 128000 \
--dbsnp dbsnp_138.hg19.vcf \
--max_alternate_alleles 15 \
--genotyping_mode DISCOVERY \
-o haplotypecaller.g.vcf \
-L manifest.bed \
--interval-padding 50
```

- -R <ref>
  Argument followed by the reference genome fasta file (hg19.fa)
- -I <bam>
  Argument followed by the input bam file
- -dt <NONE/ALL_READS/BY_SAMPLE>
  Type of reads downsampling to employ at a given locus. Reads will be selected randomly to be removed from the pile based on the method described here.
- --emit-ref-confidence <NONE/GVCF/BP_RESOLUTION>
  Argument describing the reference confidence mode. If none, regular calling is performed without emitting reference confidence calls. If GVCF, the reference model is emitted with condensed non-variant blocks. If BP_RESOLUTION, the reference model is emitted site by site. **--variant_index_type LINEAR** and **–variant_index_parameter 128000** arguments with those exact values are required if in GVCF mode.
- --dbsnp <vcf>
  dbsnp_138.hg19.vcf database file
- --max_alternate_alleles <INT>
  Maximun number of alternate alleles to genotype
- --genotyping_mode <DISCOVERY/GENOTYPE_GIVEN_ALLELES>
  Specifies how to determine the alternate alleles to use for genotyping
- -o <g.vcf>
  Argument followed by the output variant calling file with .g.vcf extension
- -L <bed>
  Argument followed by the interval list file with .bed extension
- --interval-padding <INT>
  Amount of padding (in bp) to add to each interval.

```
java -jar GenomeAnalysisTK.jar -T GenotypeGVCFs \
-R hg19.fa \
--dbsnp dbsnp_138.hg19.vcf \
--variant haplotypecaller.g.vcf \
-o haplotypecaller.vcf \
-L manifest.bed \
--interval-padding 50
```

- -R <ref>
  Argument followed by the reference genome fasta file (hg19.fa)
- --dbsnp <vcf>
  dbsnp_138.hg19.vcf database file
- --variant <g.vcf>
  A g.vcf file containing the variants called by HaplotypeCaller in GVCF mode
- -o <vcf>
  Argument followed by the output variant calling file with .vcf extension
- -L <bed>
  Argument followed by the interval list file with .bed extension
- --interval-padding <INT>
  Amount of padding (in bp) to add to each interval.

2. GATK UnifiedGenotyper

```
java -jar GenomeAnalysisTK.jar -T UnifiedGenotyper \
-R hg19.fa \
-I recalibrated.bam \
-o unifiedgenotyper.vcf \
--output_mode EMIT_VARIANTS_ONLY \
-L manifest.bed \
--interval-padding 50
```

- -R <ref>
  Argument followed by the reference genome fasta file (hg19.fa)
- -I <bam>
  Argument followed by the input bam file
- -o <vcf>
  Argument followed by the output variant calling file with .vcf extension
- --output_mode <EMIT_ALL_CONFIDENT_SITES/EMIT_VARIANTS_ONLY/EMIT_ALL_ACTIVE_SITES>
  Specifies which type of calls we should output. If set to EMIT_ALL_CONFIDENT_SITES, produces calls at variant sites and confident reference sites. IF set to EMIT_VARIANTS_ONLY, produces calls only at variant sites. If set to EMIT_ALL_ACTIVE_SITES, produces calls at any region over the activity threshold regardless of confidence. On occasion, this will output HOM_REF records where no call could be confidently made. This does not necessarily output calls for all sites in a region. This argument is intended only for point mutations (SNPs); it will not produce a comprehensive set of indels.
- -L <bed>
  Argument followed by the interval list file with .bed extension
- --interval-padding <INT>
  Amount of padding (in bp) to add to each interval.

3. SAMtools mpileup/call

```
bcftools mpileup -Ou -f hg19.fa -R manifest.bed recalibrated.bam | bcftools call -Ou -mv >
mpileup.vcf
```

- -Ou
  Argument used when piping between bcftools subcommands to speed up performance by removing unnecessary compression/decompression and VCF↔BCF conversion
- -f <ref>
  Argument followed by the reference genome fasta file (hg19.fa)
- -R <bed>
  Argument followed by the interval list file with .bed extension
- -m
  Argument set for multiallelic calling
- -v
  Argument set to output variant sites only

Functional Annotation

```
perl table_annovar.pl input.vcf humandb/ -buildver hg19 -out output.annovar -remove -
protocol refGene,cytoBand,exac03,avsnp147,dbnsfp33a,clinvar_20190305 -operation g,r,f,f,f,f
-nastring . -vcfinput
```

- /humandb <dir>
  Argument followed by the humandb directory of ANNOVAR in which variant databases are downloaded
- -buildver <hg19>
  Argument followed by the version of the human reference genome used
- -out <annovar>
  Argument followed by the ANNOVAR output file with .annovar extensions
- -remove
  Argument set to remove all temporary files
- -protocol
  Argument followed by all the protocols/databases used by the ANNOVAR script
- -operation <g/gx/r/f/>
  Argument which tells ANNOVAR what operations to used for each protocol. g means g-based, gx means gene-based with cross-reference annotation, r means region-based and f means filter-based.
- -nasting <.>
  Argument followed by a period. The period denotes a variant which does not have a score.
- -vcfinput
  Argument set to denote that the input variant file is in a .vcf format
